# Recent Advances and Remaining Challenges in Perovskite Solar Cell Components for Innovative Photovoltaics

**DOI:** 10.3390/nano14231867

**Published:** 2024-11-21

**Authors:** Pari Baraneedharan, Sankar Sekar, Silambarasan Murugesan, Djaloud Ahamada, Syed Ali Beer Mohamed, Youngmin Lee, Sejoon Lee

**Affiliations:** 1 Centre of Excellence in Photonics and Nanotechnology Research, Saveetha Engineering College, Thandalam, Chennai 602 105, India; pbaraneedharan@saveetha.ac.in (P.B.); silambarasanm@saveetha.ac.in (S.M.); 2 Division of System Semiconductor, Dongguk University, Seoul 04620, Republic of Korea; sanssekar@dongguk.edu; 3 Quantum-Functional Semiconductor Research Center, Dongguk University, Seoul 04620, Republic of Korea; 4 Department of Material Science, Central University of Tamil Nadu, Thiruvarur 610 005, India; djaloud22@students.cutn.ac.in

**Keywords:** perovskite, solar cell, hole transport layer, electron transport layer, counter electrode

## Abstract

This article reviews the latest advancements in perovskite solar cell (PSC) components for innovative photovoltaic applications. Perovskite materials have emerged as promising candidates for next-generation solar cells due to their exceptional light-absorbing capabilities and facile fabrication processes. However, limitations in their stability, scalability, and efficiency have hindered their widespread adoption. This review systematically explores recent breakthroughs in PSC components, focusing on absorbed layer engineering, electron and hole transport layers, and interface materials. In particular, it discusses novel perovskite compositions, crystal structures, and manufacturing techniques that enhance stability and scalability. Additionally, the review evaluates strategies to improve charge carrier mobility, reduce recombination, and address environmental considerations. Emphasis is placed on scalable manufacturing methods suitable for large-scale integration into existing infrastructure. This comprehensive review thus provides researchers, engineers, and policymakers with the key information needed to motivate the further advancements required for the transformative integration of PSCs into global energy production.

## 1. Introduction

With the growth in the global population and continuous advances in technology, main concerns have arisen about the reliability of the energy supply. Based on an analysis of the daily energy usage of countries such as the USA, China, and India, it has been concluded that there is a need to transition to an infinite and sustainable source of energy [1]. Energy can be obtained from primary sources such as fossil fuels (e.g., coal, petroleum, and natural gas), combustion, and nuclear fission, or transformed into secondary forms such as electricity, hydrogen, compressed air, and microwave radiation. However, conventional energy sources have a deleterious effect on the environment, directly polluting local environments and contributing to climate change [2,3]. To address this degradation of the environment, there has been a global shift from fossil fuels to renewable energy sources [4] such as solar energy, which represents a viable solution to potential energy shortfalls due to its reliability, environmental friendliness, and accessibility.

Solar energy as a method of energy generation for industrial use originates from the observation by Alexandre Edmond Becquerel in 1839 of the production of photo-voltage from two similar electrodes in a dilute acid electrolyte under illumination [5]. In 1877, Prof. W. G. Adams and R. E. Day conducted experiments on the electrical behavior and sensitivity of solidified selenium (Se) following contact with light. They concluded that a potential difference could be produced around the molecule, inducing light sensitivity in annealed Se [6]. In 1883, American Charles Fritts invented the first Se solar cell by coating it with a thin layer of gold (Au) metal. The efficiency of the cell was less than 1% [7]. Subsequently, in 1904, Wilhelm Hallwachs used copper (Cu) and copper oxide (CuO_x_) to construct a solar cell. In 1954, Bell Labs applied a single-crystal silicon (Si) solar cell for the first time, with an estimated efficiency of 6%. 

Solar cells are semiconducting devices with a p-n junction that utilize the photovoltaic effect to generate electricity. The industrial market for solar cells is dominated by three generations of manufacturing systems. The first generation consists of Si wafer-based solar cells made of monocrystalline or polycrystalline Si and gallium arsenide (GaAs) semiconductor materials. Although high-quality Si solar cells are highly efficient and durable, their raw materials and purification processes are expensive. The second generation of solar cells uses thin-film technology to reduce the bulk materials to a layer with a thickness of a few hundred nanometers to a few micrometers, and thus reduce manufacturing costs. Hydrogenated amorphous Si, cadmium telluride, and copper indium gallium diselenide are commonly employed for second-generation solar cells [8]. Third-generation solar cells are still in the early stages of development and include dye-sensitized solar cells (DSSCs), organic and polymeric solar cells, quantum dot (QD) solar cells, perovskite solar cells (PSCs), and multi-junction solar cells [9,10,11]. Among them, PSCs have received particular attention due to their ease of acquisition and advanced properties, improving device performance and exhibiting greater potential for commercialization on a large scale with lower environmental risks. As assembled upon the unique crystal structure of perovskite materials, which commonly incorporate organic–inorganic hybrid halide compounds, PSCs have transformed solar cell research in recent years [12,13,14]. This breakthrough is largely due to their adjustable bandgaps, strong light absorption, and the simplicity of production through low-temperature solution processes. These characteristics enable the development of lightweight, flexible, and efficient solar modules, ideal for applications where traditional rigid silicon cells may be less suitable. Since their initial efficiency of around 3.8% was recorded in 2009, PSCs have achieved significant progress, with current efficiencies surpassing 25%, making them strong contenders against silicon-based technologies [15,16]. Despite these remarkable advancements, PSCs are still facing several significant hurdles, particularly concerning their long-term stability and environmental impact. Another promising research direction is the development of PSC materials for tandem solar cells, which integrate multiple absorber layers with varying bandgaps to capture a wider range of the solar spectrum, boosting overall efficiency. When combined with silicon or other thin-film technologies, perovskite tandem cells have achieved efficiencies that surpass those of single-junction cells, underscoring their potential to break new ground in photovoltaic efficiency [17,18,19,20]. However, significant technical and manufacturing challenges must be addressed before these innovations can be commercially viable. Specifically, creating stable hole transport layers (HTLs) and electron transport layers (ETLs), as well as scalable manufacturing methods, is essential for bringing PSCs to market. Recent studies are exploring various organic and inorganic materials to improve the interface stability and overall lifespan of PSC devices [21,22,23]. The rapid advancements in PSC research highlight the importance of addressing existing challenges to move this technology closer to large-scale deployment. Additionally, the development of sustainable and cost-efficient manufacturing processes is crucial to meeting the environmental goals associated with renewable energy solutions. To appropriately address these issues, researchers are exploring various approaches, such as affordable encapsulation techniques and alternative deposition methods, positioning PSCs as a strong contender for next-generation photovoltaic applications [24,25,26,27].

Due to the transformative potential of PSCs for renewable energy systems, this review seeks to summarize recent innovations in PSC components, including absorber layers, transport layers, and interface materials. The intrinsic advantages of perovskite materials include high light-absorbing properties, ease of fabrication, and potential for low-cost production, but the widespread adoption of PSCs is limited by a general lack of stability, scalability, and efficiency. This review thus aims to highlight recent breakthroughs in the development of perovskite materials with improved stability and scalability. By exploring innovations in absorber layer engineering, ETLs, HTLs, and interface materials, the present review will help understand how each of these components influences the overall performance of PSCs.

## 2. Perovskite Crystal Structure

**Figure 1:**
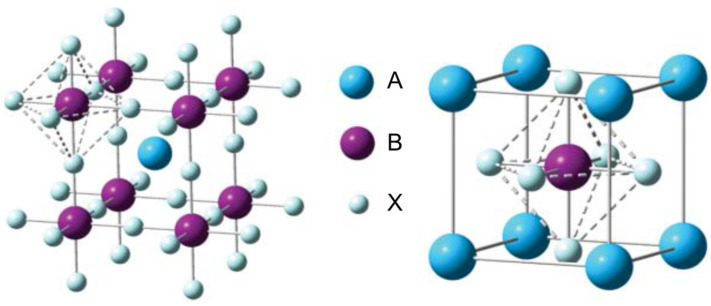
Cubic lattice of the perovskite crystal ABX_3_. Reproduced with permission [31], Copyright (2021) by John Wiley and Sons.

Perovskite was first discovered by German mineralogist Gustav Rose in 1839 and named after Lev Perovski in recognition of his outstanding contributions to mineralogy [28,29]. Perovskite is a subclass of the mineral compound calcium titanate (CaTiO_3_) and can generally be expressed as ABX_3_, where A represents a monovalent organic cation such as methylammonium (CH_3_NH_3_^+^, MA^+^), formamidinium ((CH(NH_2_)_2_^+^, FA^+^), and ethylammonium (CH_3_CH_2_NH_3_^+^, EA^+^) or an inorganic cation such as potassium (K^+^), rubidium (Rb^+^), and cesium (Cs^+^), while B is a divalent metallic cation that can include elements such as lead (Pb^2+^), tin (Sn^2+^), and germanium (Ge^2+^), and X is a halogen ion such as iodine (I^−^), bromine (Br^−^), and chlorine (Cl^−^) or a group of halogen anions [30]. The A-site cation of a perovskite is larger than the divalent B-site cation and is linked to a 12-fold coordination site with the halide anions, resulting in an AX_12_ cuboctahedron. The B-site cation, on the other hand, is associated with six-fold coordination with the halide anions and is surrounded by a BX_6_ octahedron, as illustrated in Figure 1.

The stability, equilibrium, and formability of a perovskite are strongly influenced by both the A- and B-site cations. Several recent reports have suggested that, although the A-site cation plays no role in the optoelectronic properties, it serves as a regulator of the lattice structure, and thus influences its stability [32]. To predict the stability and morphological properties of a perovskite, two factors need to be considered: the Goldschmidt tolerance factor (*t*; Equation (1)) and the octahedral factor (*µ*; Equation (2)):

(1) $$t=\frac{R_{a}+R_{x}}{\sqrt{2}(R_{b}+R_{x})}$$

(2) $$\mu =\frac{R_{a}}{R_{x}}$$

where *R_a_*, *R_b_*, and *R_x_* are the ionic radii of the constituent ions A, B, and X, respectively. The crystal structure of the perovskite is stable when the tolerance factor is around 0.9, while an ideal cubic lattice is formed when *t* equals 1. In certain cases, the perovskite lattice can deviate from its normal morphology and form a distorted structure, typically an orthorhombic or tetragonal crystal lattice when *t* is less than 0.8 or a non-perovskite structure when *t* is greater than 1. For example, structures such as hexagonal or ilmenite-type FeTiO_3_ are formed. On the other hand, the octahedral factor is used to determine the extent of the stability of the [BX_6_] cuboctahedron. Empirical studies have shown that a value of 0.414 or higher results in a stable perovskite structure [33]. However, if there is a slight discrepancy in the size of the A cation, the tolerance factor becomes abnormal, and the [BX_6_] cuboctahedron tilts to compensate for this, resulting in a non-perovskite or a distorted morphology that impacts the structure of the perovskite. The various factors associated with the perovskite structural components are summarized in Table 1.

## 3. Perovskite Absorbing Layers

**Figure 2:**
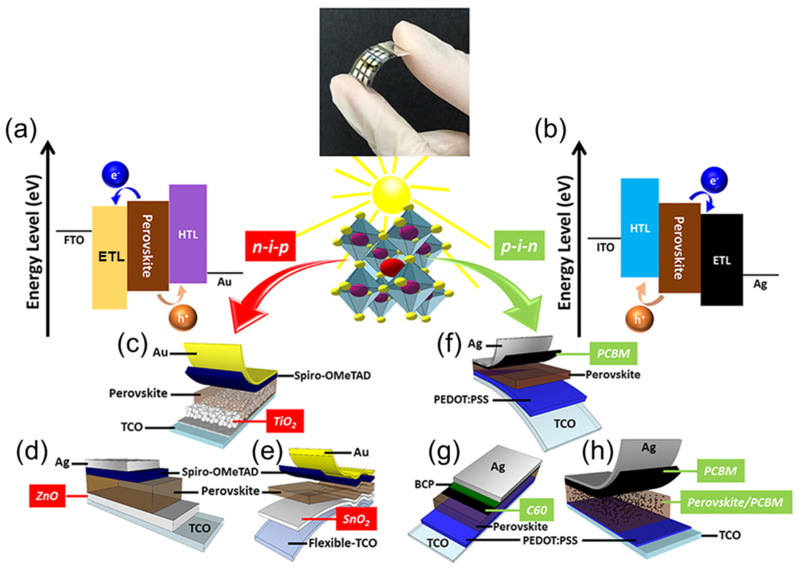
Evolution of PSCs: (**a**,**b**) energy levels of PSCs and (**c**–**h**) device structures of PSCs. Reprinted from [37], Copyright (2019) by Frontiers Group.

PSCs are viewed as an extension of Grätzel cells, which were initially developed for oxide applications (Figure 2) before Miyasaka et al. replaced the organic compound titanium dioxide (TiO_2_), which was used as the visible-light sensitizer in DSSCs, with the halide compounds of methylammonium lead iodide (CH_3_NH_3_PbI_3_) and methylammonium lead bromide (CH_3_NH_3_PbBr_3_) in a liquid electrolyte. The resulting devices had efficiencies of 3.13% and 3.81% for CH_3_NH_3_PbBr_3_ and CH_3_NH_3_PbI_3_, respectively [35,36].

To improve the performance of solar cells using organo-lead halide absorbing layers, the same team substituted the electrolyte HTL with the solid-state HTL 2,2′,7,7′-tetrakis [N,N-di(4-methoxyphenyl) amino]-9,9′-spirobifluorene (Spiro-OMeTAD) using the same process and achieved an efficiency of 10% [38]. The photoanode in a DSSC can be likened to the absorbing layer in a PSC, which absorbs light and generates electron–hole pairs or excitons. Because of the low binding energy of these excitons within the perovskite, they can split into pairs of electrons and holes that move through the network to produce an electric current [39]. However, defects between layers and other imperfections can lead to losses such as recombination, thermalization, and optical or special relaxation loss. There are many intrinsic and extrinsic factors that can reduce the performance of the absorbing layer, including the properties of the materials used, which can result in energy losses, and the use of thin-film deposition to fabricate the active layer [40].

### 3.1. Chalcogenide Perovskites-Based Absorbing Layers

**Figure 3:**
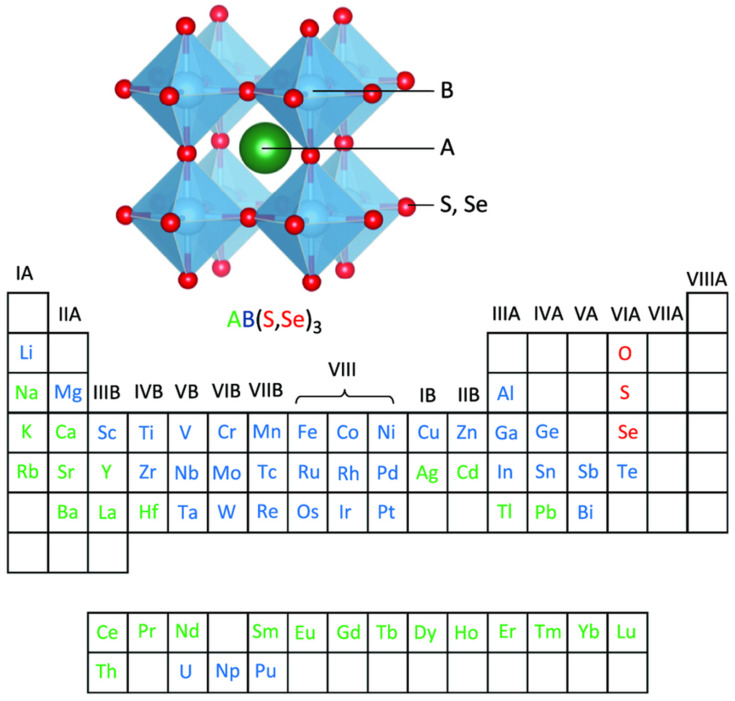
Chalcogenide perovskite lattice and chemical elements at sites A and B. Reproduced with permission [48], Copyright (2019) by John Wiley and Sons.

Chalcogenide perovskites were first synthesized in 1950, but it was not until 2015 that they were explored as potential solar cell materials [41]. These semiconductors have a higher electronegativity than conventional semiconductors and have properties that are more similar to halide perovskites than oxide materials [42]. Despite being studied for their crystalline and atomic structures, their physical properties were not well-known until the use of first principle calculations and density functional theory characterization [43]. The evaluation of around 18 ABX_3_ chalcogenides revealed promising properties such as a high electron mobility, a direct band gap, and high-quality optical absorption, making them potential candidates for optoelectronic and photovoltaic applications [44]. Subsequent research has focused on revealing the physical, chemical, and magnetic properties of chalcogenide perovskites so that they can be used in cutting-edge devices [45]. As shown in Figure 3, the A-site element is typically an alkaline metal, but some *d*-block and *f*-block transition metals with a 2^+^ oxidation state can also be used. The B-site element can be a tetravalent element with a 4^+^ oxidation state, such as Ti, Zr, or Hf, as well as some elements from the *d*-, *p*-, and *f*-blocks. Chalcogenide perovskites exhibit a variety of structural motifs and bonding modes with the octahedral BX_6_ unit, and only a few of these structures conform to the perovskite criteria for the tolerance and octahedral factors. However, the bonding rules from halides are still applicable to chalcogenide perovskites. In the lattice structure of chalcogenide perovskites, six X-site anions are connected to the B-site cations, resulting in the formation of [BX_6_]^n−^ octahedra that are covalently bonded to their respective corners and dictate the electrical and optical properties of the chalcogenide perovskite [46,47].

### 3.2. Organic–Inorganic Hybrid Perovskite Absorbing Layers

The synthesis of organic–inorganic hybrid perovskites (OIHPs) can be traced back to the work of Christian Møller, a Danish scientist who, in 1882, synthesized cesium lead halide (CsPbX_3_, X = I, Br, Cl) from a solution [49]. The first attempt at synthesizing a perovskite absorbing material was made by the French researcher Jacques-Joseph Ébelmen in 1851, who used the flux growth method to prepare the mineral compound calcium titanate (CaTiO_3_) [50]. In 1978, Dieter Weber made a ground-breaking contribution by replacing the monovalent inorganic cation Cs with the small cation methylammonium (CH_3_NH_3_^+^) in a lead halide compound, thus leading to the development of the first OIHP. Recently, there has been renewed interest in the use of OIHPs in various academic and industrial applications [51]. In 2009, Kojima et al. made a significant discovery when they incorporated the halide compounds CH_3_NH_3_PbI_3_ and CH_3_NH_3_PbBr_3_ in a DSSC with the aim of enhancing the dye-sensitized device in a liquid electrolyte. 

This breakthrough led to a new era of research in perovskites, with approximately 61,700 citations on Google Scholar since 2009, demonstrating the considerable interest in their exceptional sustainability and longevity, which are inherent properties of their structure and composition [52]. Methylammonium lead iodide (CH_3_NH_3_PbI_3_) is a notable OIHP with a band gap of 1.55 eV and a cubic crystal structure at high temperatures above 330 K. As the temperature changes, it undergoes a phase change to tetragonal and orthorhombic structures [53,54]. Methylammonium lead iodide has been utilized in various optoelectronic applications such as light-emitting devices, lasers, photodetectors, phototransistors, field-effect transistors, and photovoltaics. Its ferroelectricity, direct bandgap, high mobility, low efficient mass, and low trap state density lead to the efficient emission of light and the conservation of particle energy without a change in momentum between bands. Although methylammonium (CH_3_NH_3_^+^, MA^+^) is the most widely used small organic A-site cation, other larger organic cations have been found to be effective in maintaining the charge equilibrium and enhancing stability while slightly reducing the optical band gap, thus increasing the range of the solar spectrum that can be absorbed by the perovskite. For example, in 2014, Eperon et al. substituted methylammonium with formamidine (CH(NH_2_)_2_, FA) to synthesize formamidine lead iodide (CH(NH_2_)_2_PbI_3_), which exhibited considerable band gap tunability and an efficiency of 14%. 

Interface passivation engineering is a highly successful method for improving the efficiency and stability of PSCs. It involves defect passivation, which minimizes charge recombination, prevents ion migration, and helps in controlling hysteresis. Interface passivation layers can be created using solution and/or vacuum evaporation techniques. These methods are highly flexible and can be applied to a wide range of materials with diverse characteristics and fabrication procedures. This leads to improved photovoltaic performance and stability and opens new possibilities for improving the performance of the light-absorbing layer [54].

**Figure 4:**
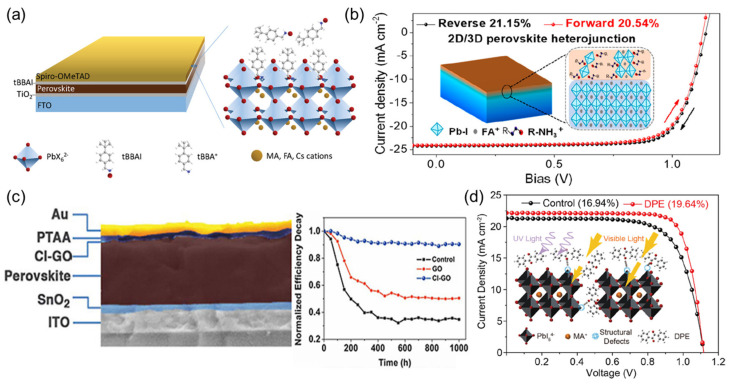
Passivation engineering: (**a**) reducing trap states to improve performance, (**b**) removal of hysteresis, (**c**) enhancing stability, and (**d**) photosensitive molecule-assisted passivation to inhibit undesired defect-assisted recombination. (**a**) Reprinted from [54], Copyright (2021) by Elsevier’s Group. (**b**) Reproduced with permission [55], Copyright (2019) by American Chemical Society. (**c**) Reprinted from [56], Copyright (2021) by RSC Group. (**d**) Reproduced with permission [57], Copyright (2023) by American Chemical Society.

Alternative organic cations to methylammonium and formamidine have also been investigated for use in OIHPs. Guanidinium, for example, has been found to be a useful doping agent when used in conjunction with other organic cations. However, its large size makes it unsuitable as a replacement for the A-site cation in the perovskite structure. Nevertheless, it has been successfully used as a passivating agent to improve the quality and efficiency of perovskite films (Figure 4). Photosensitive molecule-assisted passivation efficiently prevents undesirable defect-assisted recombination, increasing the power conversion efficiency (*PCE*) from 16.94% to 19.64%. Passivated devices demonstrate significantly improved stability under light, with >80% of the original *PCE* preserved after 700 h. This method helps to create defect-free and UV-resistant perovskite-based photoactive layers for highly efficient and stable PSCs.

The B-site cation covalently bonds with the halide anion X and influences the stability of the BX_6_ octahedron via the octahedral factor, which is the ratio of the ionic radii of the B-site cation and the halide anion. This is essential for evaluating the charge distribution within the optical band gap of the perovskite. Pb, a metallic element from group 14 of the periodic table, is the most widely used element for bonding with the halide anion. However, tin (Sn) and germanium (Ge) have also been used in the B-site position to mitigate the environmental impact of the excessive use of Pb because they share almost the same properties and belong to the same group. As shown in Table 2, tandem structures integrate multiple perovskite layers, optimizing absorption across the solar spectrum, while 2D/3D hybrid configurations combine organic and inorganic perovskites to enhance their stability. In addition, QDs and nanostructures tailor light absorption, while perovskite–Si tandem cells leverage the strengths of both materials. Light-harvesting innovations enable PSCs to excel in various environmental conditions, which is important for sustainable energy solutions.

### 3.3. Copper-Zinc-Tin-Sulfides/Selenide (CZTS/CZTSe) Based Absorber Materials

Copper–zinc–tin–sulfides/selenide (CZTS/CZTSe) absorber materials have attracted considerable interest in recent years owing to their potential for economical, sustainable, and high-efficiency solar cells. These materials are part of the kesterite family and are increasingly regarded as viable alternatives to copper–indium–gallium–selenide (CIGS) in thin-film photovoltaic applications. CZTS and CZTSe possess numerous significant benefits over CIGS, particularly due to their composition of abundant, non-toxic, and cost-effective materials, rendering them economically and environmentally suitable for large-scale solar applications.

The kesterite crystal structure of CZTS and CZTSe, formed by tetrahedral coordination of cations with sulfur or selenium, supports high optical absorption (∼10^4^ cm^−1^) across the solar spectrum, making them suitable for absorbing layers as thin as 1–2 µm. The bandgap tenability, 1.5 eV for CZTS and 1.0 eV for CZTSe, enables optimal utilization of solar photons and allows for efficient charge separation, positioning these materials in the ideal energy range for single-junction photovoltaic cells. Advanced characterization techniques, including synchrotron-based X-ray diffraction and Raman spectroscopy, reveal that kesterite structures exhibit complex defect formations, such as antisite defects and vacancies, which influence electronic properties. Optimizing these defects through precise control of stoichiometry and fabrication conditions remains critical for advancing CZTS/CZTSe efficiency [82,83].

A variety of fabrication methods have been explored to optimize CZTS/CZTSe absorber layers, each of which offers specific benefits for controlling material composition and morphology. Techniques such as co-evaporation, magnetron sputtering, and electrochemical deposition provide high control over stoichiometry but involve vacuum processes that add to manufacturing costs. Alternatively, solution-based methods, including sol–gel, hydrazine-based, and nanoparticle inks, are cost-effective and scalable for industrial applications. However, solution-based synthesis introduces challenges related to phase purity, as secondary phases (e.g., ZnS, SnS, Cu_2-x_S) often form and impede device performance. Advances in hybrid methods combining solution deposition with subsequent annealing in controlled atmospheres have shown promise in improving phase purity and enhancing grain growth, thereby reducing recombination losses and improving device efficiency [84,85].

#### 3.3.1. Comparative Evaluation of CZTSe/CZTS and CIGS Absorber Materials 

CZTS/CZTSe outshines CIGS in terms of material abundance and cost, given the scarcity and expense associated with indium and gallium. The environmental profile of CZTS/CZTSe is favorable as they lack toxic elements like cadmium and lead, often present in other semiconductor materials, thereby minimizing ecological impact over the lifecycle of the solar cell. This eco-friendly aspect makes them more appealing for applications where regulatory constraints on hazardous materials are in place [86,87]. In terms of efficiency, CIGS still maintains an edge due to its superior crystallinity and fewer defect states. However, with recent advances in grain boundary passivation and defect engineering, CZTS/CZTSe cells are beginning to close the efficiency gap, reaching efficiencies upwards of 12%.

#### 3.3.2. Current Challenges and Mitigation Strategies

Key challenges for CZTS/CZTSe absorbers include defect-related losses, phase segregation, and poor charge carrier transport due to cation disorder and secondary phase formation. Recent studies have demonstrated that alkali metal doping (e.g., Na, K) and post-deposition selenization/sulfurization treatments can enhance grain growth and passivate surface and grain boundary defects, thereby reducing recombination sites and improving open-circuit voltage. Passivation layers such as TiO_2_ or Al_2_O_3_ have also been implemented as effective surface treatments to suppress carrier recombination and stabilize interface properties. Additionally, tandem cell architectures that incorporate CZTS/CZTSe as top cells in multi-junction configurations are being investigated to overcome the bandgap limitations of single-junction devices and improve overall conversion efficiencies. The efficiency roadmap for CZTS/CZTSe solar cells is promising, particularly with ongoing research focusing on passivation, alloying, and advanced device architectures. Efforts are underway to achieve commercial viability by addressing the performance stability and efficiency limitations through refined synthesis protocols and defect passivation strategies. Tandem structures combining CZTS/CZTSe with perovskites or other wide-bandgap absorbers hold potential for surpassing single-junction efficiency limits, bringing CZTS/CZTSe-based devices closer to market readiness [16,88,89].

## 4. Hole Transport Layers (HTLs)

HTLs are semiconductor materials consisting of either pure or compound elements in which most of the carriers are positively charged particles. In 2012, Park et al. first used Spiro-OMeTAD as an HTL in a dye-sensitized device, replacing the liquid electrolyte-based HTL to improve device performance. The use of the HTL in a PSC serves multiple purposes, including collecting and transporting positively charged holes to the metal electrode, preventing negatively charged electrons from accessing the anode, and aiding in the separation of charge carriers after excitation due to photon absorption by the active layer, with sufficient energy equal to or greater than the band gap of the light-absorbing material in the conventional n-i-p configuration. 

An ideal HTL for a PSC must have a number of important characteristics, including (a) high mobility of charge carriers and material conductivity, (b) a matching band alignment with the active layer for easy transfer of particles, (c) a high low unoccupied molecular orbital (LUMO) to block the passage of electrons to the positive electrode, (d) thermal, moisture, UV, and chemical stability, (e) a large optical bandgap to intercept the electromagnetic radiation spectrum, (f) a hydrophobic nature, and (g) affordable fabrication methods [90]. The HTL acts as a barrier between the active layer and the metal electrode, reducing recombination and improving device stability and the open-circuit voltage (*V_oc_*) [91]. In addition, the highest occupied molecular orbital (HOMO) energy level should be close to the valence band of the perovskite absorbing material [92].

### 4.1. Organic HTLs

The molecular structure of HTLs consists of an aromatic compound core that is linked to many electrons [93]. The HTL is essential for achieving maximum efficiency and device reproducibility, and its role is critical in PSCs, depending on the type of configuration that is employed. This section focuses on organic HTLs that are widely used and new materials that are in the initial stages of development.

#### 4.1.1. Spiro-OMeTAD

**Figure 5:**
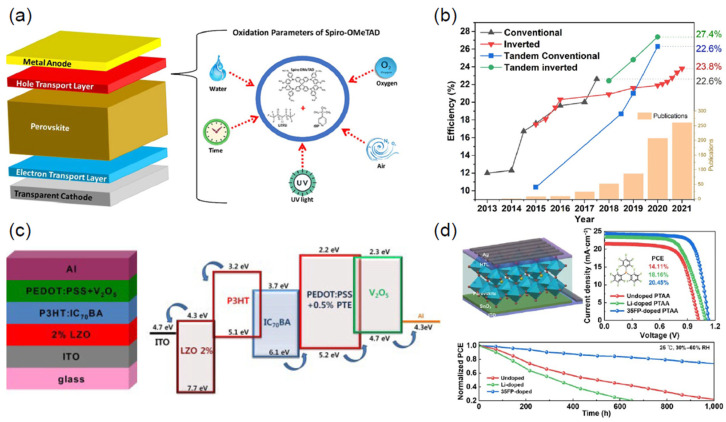
(**a**) Oxidation parameters for Spiro-OMeTAD-based PSCs. (**b**) Band structure and energy level of an inverted organic photovoltaic device that utilizes an HTL of PEDOT:PSS doped with V_2_O_5_. (**c**) Examples of PTAA used with a perovskite and the resulting efficiency. (**d**) Doped PTAA with improved charge transport properties and a lower trap density. (**a**) Reproduced with permission [94], Copyright (2022) by American Chemical Society. (**b**) Reprinted [95], Copyright (2021) by John Wiley and Sons. (**c**) Reproduced with permission [96], Copyright (2014) by American Chemical Society. (**d**) Reproduced with permission [97], Copyright (2022) by Springer Nature Group.

Spiro-OMeTAD is a highly efficient HTL commonly used in the planar (n-i-p) PSC configuration. The compound was first synthesized in 1997 by Selbeck’s group, who added an electron-donating p-methoxy group to the Spiro-TAD molecule. Spiro-OMeTAD has a wide range of applications as a p-type semiconducting material for photo-operating devices. In 2015, Jaramillo-Quintero et al. used Spiro-OMeTAD as the hole-injecting layer in a light-emitting diode (LED), resulting in a highly satisfactory performance compared to previous reports. Spiro-OMeTAD has achieved remarkable performance and exhibits a high *PCE* in solar cells, especially after the substitution of the liquid electrolyte with a solid-state electrolyte in DSSCs. Its excellent alignment with the HOMO and LUMO energy levels of the state-of-the-art perovskite CH_3_NH_3_PbI_3_ has motivated its widespread adoption. This led to it breaking the record for efficiency in the last two decades, with a *PCE* of 25.21% reported in 2021. However, due to its pseudo-amorphous nature, Spiro-OMeTAD requires several oxidation processes, including doping (Figure 5), to enhance charge mobility. The doping agent can migrate to the perovskite absorbing layer, reducing the stability of the PSC.

#### 4.1.2. Poly[bis(4-phenyl)(2,4,6-trimethylphenyl) Amine (PTAA)

Thelakkat et al. [98] employed poly[bis(4-phenyl)(2,4,6-trimethylphenyl) amine (PTAA), a p-type organic semiconductor polymer synthesized through the Ullmann reaction in 1999, as a hole-injection layer and an HTL in organic LEDs. PTAA can be deposited at room temperature and has advantageous properties, encouraging researchers to explore its use in both conventional and inverted planar configurations as a charge transport layer (CTL). With the use of organic doping agents such as tBP and LiTFSI salt, PTAA exhibits higher charge mobility (4 × 10^−3^ cm^2^ V^−1^ S^−1^) than Spiro-OMeTAD (2 × 10^−4^ cm^2^ V^−1^ S^−1^), resulting in the significant suppression of charge recombination and an increase in *V_oc_* and the fill factor (*FF*) in both conventional and inverted planar configurations. 

The hydrophilic nature of commonly used organic HTL dopants raises concerns about their impact on the lifespan of perovskites. To address this, hydrophobic organic dopants are required. Chao Yu and colleagues utilized tris(pentafluorophenyl)phosphine (35FP) as an organic dopant instead of tBP and LiTFSI, resulting in a significant improvement in the longevity and performance of the PSC, with the total *PCE* improving by 20.45% compared to undoped PTAA (14.11%) [97]. Another breakthrough in the use of PTAA with perovskites is the low-temperature deposition process (Figure 5), which prevents damage to other parts of the perovskite and leads to high efficiency, especially when using the inverted (p-i-n) configuration rather than the conventional (n-i-p) configuration.

#### 4.1.3. Poly(3,4-ethylenedioxythiophene)-poly(styrene sulfonate) (PEDOT:PSS)

Poly(3,4-ethylenedioxythiophene)-poly(styrene sulfonate) (PEDOT:PSS) is commonly used as an HTL in the planar inverted perovskite configuration (p-i-n) due to its ease of preparation and band alignment with the perovskite, which facilitates hole migration to the anode and prevents the movement of electrons to the counter electrode [99,100]. Researchers are interested in exploring its applications in various optoelectronic devices because of its ability to deal with electromagnetic radiation and its smooth surface on an indium tin oxide (ITO) substrate. The use of organic HTL compounds helps to reduce defects that affect the performance of PSCs and prevent hysteresis, which can compromise the stability of the device over time. However, PEDOT:PSS has some disadvantages, such as its acidic nature due to the PSS chain, which can lead to the degradation of the perovskite and the overlying electrode. It also has a low *V_oc_* and short-circuit current (*J_sc_*), resulting in a weak *PCE*. Additionally, questions remain about the stability of PEDOT:PSS-based devices due to the hygroscopicity of the organic polymer [101]. To address these issues, various technical strategies have been developed to replace PEDOT:PSS or minimize its negative impact on device performance. As shown in Figure 5, Lee and his team used vanadium peroxide (V_2_O_5_) as a doping agent in 2015 and observed an improvement in device performance, with the *PCE* increasing from 4.04% to 4.40%. The V_2_O_5_-doped PEDOT:PSS HTL was stable in air and served as the interfacial layer, contributing to the excellent stability of the device [96]. Graphene oxide has also been used instead of the acidic PSS chain (PEDOT:GO) in an inverted PSC, resulting in an efficiency of 8.21%, while also suppressing exciton quenching and eliminating the recombination process at the HTL/active layer interface [102]. In addition, the acidic PSS chain was replaced with soluble lignosulfonate in 2015, resulting in an efficiency of 5.79% [103].

ZnO is a widely used ETL in organic solar cells (OSCs) due to its good electron mobility and ease of solution processing. However, ZnO surfaces often contain defects, such as oxygen vacancies, which can lead to charge recombination at the interface with the active layer, thereby limiting device efficiency. Introducing controlled defects through gamma irradiation can improve the ZnO’s crystalline quality and reduce defect density, which subsequently enhances charge extraction and transfer characteristics [14,104]. In recent experiments, a gamma-ray-assisted solution process has been developed for ZnO ETL preparation, where gamma irradiation alters ZnO nanoparticles directly within the precursor solution [105]. This method creates ZnO films with substantially fewer intrinsic defects compared to conventional ZnO films. In OSCs, gamma-ray-treated ZnO (ZnO-G) films demonstrate a remarkably high *PCE*, reaching 11.09%. This enhancement is largely due to the improvement in *V_oc_*, *J_sc_*, and *FF* in devices based on ZnO-G ETLs, attributable to enhanced charge collection efficiency and reduced charge recombination [106]. The gamma ray process effectively passivates surface defects on ZnO, mitigating the issues associated with oxygen vacancies and hydroxyl groups on the ZnO surface. These vacancies typically serve as recombination centers, so their passivation via gamma irradiation leads to increased carrier lifetimes and more effective charge transport across the ETL-active layer interface. This defect engineering method thus reduces recombination, allowing for more efficient charge extraction, which directly contributes to the higher *PCE*, and improved stability observed in gamma-irradiated ZnO ETL-based devices. Incorporating gamma irradiation as a tool for defect engineering in HTL and ETL layers introduces an innovative approach for enhancing OSC efficiency. By controlling irradiation parameters such as dose and environmental conditions, it is possible to fine-tune the electrical properties of materials like PEDOT:PSS and ZnO, achieving a balance between increased carrier concentration and minimized recombination pathways. This interfacial optimization extends the operational lifetime of OSCs and improves stability under continuous illumination. The inclusion of defect-enhanced performance strategies in this paper broadens its impact, highlighting how ionizing irradiation can be a versatile, cost-effective solution for improving charge transport layers in OSCs. This approach appeals to a broader audience by addressing how innovative defect engineering can be leveraged to push the boundaries of organic solar technology, offering new directions for achieving stable, high-efficiency devices in a scalable, reproducible manner [107].

#### 4.1.4. Poly(3-hexylthiophene) (P3HT)

**Figure 6:**
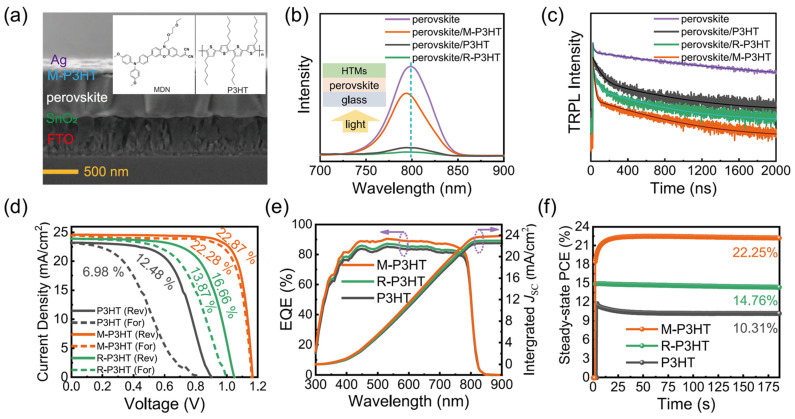
(**a**) Cross-sectional SEM image of PSC with M-P3HT. (**b**) Steady-state PL spectra. (**c**) TRPL spectra. (**d**) J–V curves of champion devices based on P3HT, M-P3HT, and R-P3HT. (**e**) EQE spectra and integrated photocurrent curves of device with P3HT, M-P3HT, and R-P3HT. (**f**) Steady-state *PCE*. Reproduced with permission [109], Copyright (2022) by Springer Nature Group.

Due to the high cost of charge transport materials, there is a need to explore alternative HTL candidates that have the necessary properties for use in PSCs. Poly(3-hexylthiophene) (P3HT), a p-type semiconductor and member of the polythiophene family, is a promising candidate that facilitates the movement of holes due to its electron-rich nature. This soluble organic semiconducting polymer has become a standard in organic electronic and solar cells as a CTL. Thiophene, the core of P3HT, was first isolated from coal tar in 1883 and has been the subject of ongoing investigation for its various properties and applications, including as a semiconducting layer in field-effect transistors due to its compatibility with glass materials. P3HT has also been compared in performance to other CTLs in DSSCs and has been utilized as a CTL in PSCs to replace more expensive materials such as Spiro-OMeTAD. However, to the best of our knowledge, this compound is rarely used in its pristine state; rather, it is often modified or blended with other carbon-based compounds to enhance its charge transport properties. The absence of HTLs in carbon-based PSCs significantly constrains further *PCE* improvements and poses a challenge to the broader commercial viability of these devices. A one-step additive-assisted strategy is used, where poly(3-hexylthiophene) (P3HT) is introduced in chlorobenzene at an optimized concentration to enhance the perovskite layer properties. The integration of P3HT into the perovskite layer notably promotes the crystallinity of the perovskite film, reducing grain boundaries and passivating defects, thereby effectively mitigating non-radiative recombination pathways. This optimized P3HT additive creates a graded perovskite/P3HT heterojunction, which enhances hole extraction efficiency, leading to substantial *PCE* improvements. Specifically, *PCE* increased from 12.72% to 15.57% in conventional devices and from 11.81% to 13.54% in flexible devices, demonstrating that our additive-assisted heterojunction approach is robust for enhancing efficiency in both rigid and flexible carbon-based PSCs without requiring additional HTLs [108]. The cross-sectional SEM image of the PSC architecture with modified P3HT (M-P3HT), shown in Figure 6, provides a detailed insight into the layer structure, displaying the active layer, ETL, and HTL, with M-P3HT employed as the HTL. The SEM image revealed efficient interfacial contact and optimal layer thickness, which are crucial for charge separation and transport [109]. The J–V characteristics of PSCs with P3HT, M-P3HT, and regio-random P3HT (R-P3HT) HTLs revealed significant performance distinctions, with the champion device composed of M-P3HT, demonstrating superior *J_sc_* and *V_oc_* compared to those with P3HT and R-P3HT. This improvement, attributed to molecular modifications in M-P3HT, facilitates better energy level alignment with the perovskite layer and enhances charge extraction. The external quantum efficiency (EQE) spectrum and integrated photocurrent curves demonstrate that the M-P3HT-based device has a broader and higher response across the visible spectrum, translating to elevated integrated photocurrent and high photon-to-electron conversion efficiency. This was also confirmed by steady-state *PCE* measurements, where the M-P3HT device maintained significantly higher efficiency compared to P3HT and R-P3HT under continuous illumination. The stability of the device at the maximum power point, under simulated full solar illumination (AM 1.5 G, 100 mW cm^−2^) in a glovebox, indicated that M-P3HT-based PSCs has greater durability, likely due to M-P3HT’s resistance to degradation under light and thermal stress, making M-P3HT a promising HTL candidate for high-efficiency, long-term photovoltaic applications [109].

### 4.2. Inorganic HTLs

**Figure 7:**
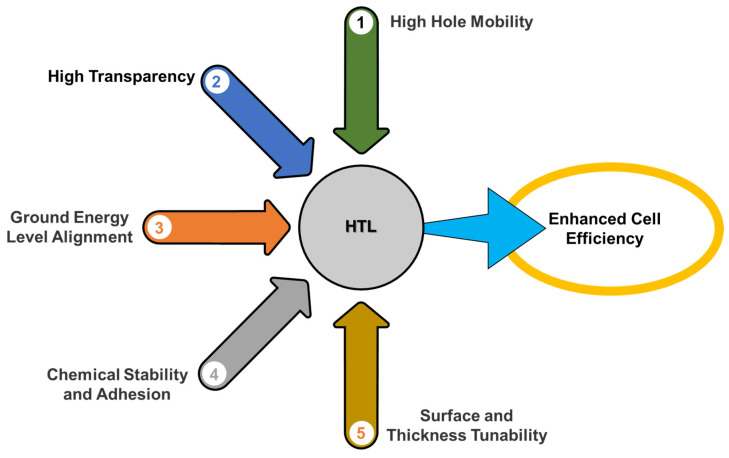
Potential approaches for the HTL to enhance PSC efficiency.

The high *PCE* of PSCs and tandem solar cells is largely due to the use of suitable CTLs that reduce the obstacles between the interfaces of different layers and promote high charge mobility, thus mitigating defects. Of the commonly used HTLs, Spiro-OMeTAD, PTAA, and other polymeric p-type semiconductors require significant modification through doping with agents such as LiTFSI and tBP, which can compromise device stability. Figure 7 illustrates the approaches used for HTL design to increase the efficiency of PSCs. Although organic HTLs are highly efficient, their purification technology is prohibitively expensive. Therefore, it is essential to consider alternative inorganic p-type semiconductors as hole carriers for PSC applications that are efficient, stable, resistant to high temperatures and moisture, and cost-effective. Metal-oxide- and sulfide-based p-type semiconductors are preferred due to their chemical stability, low-cost processing, high charge mobility, and precise band gap adjustment [110].

#### 4.2.1. Nickel Oxide (NiO_x_)

**Figure 8:**
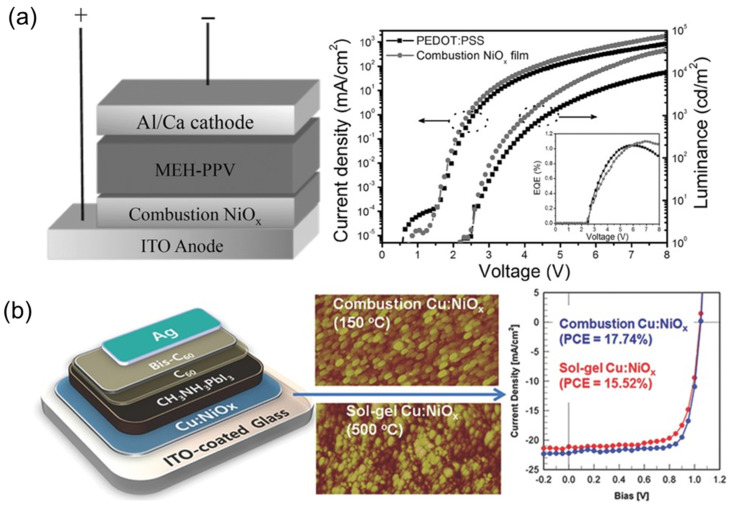
(**a**) Device structure and relationship between current density and brightness with respect to applied bias (inset: EQE curves) and (**b**) high crystallinity, conductivity, and hole-extraction properties of a PSC with high *PCE* of 17.74% using low-temperature-processed Cu-doped NiO_x_ [114,116]. (**a**) Reproduced with permission [114], Copyright (2013) by John Wiley and Sons. (**b**) Reproduced with permission [116], Copyright (2015) by John Wiley and Sons.

Due to its outstanding optoelectronic properties, including a high light transmittance in the visible and near-ultraviolet (UV) range, nickel oxide (NiO_x_) has been employed as an efficient HTL for PSC applications [111]. NiOx is a p-type semiconductor in its pristine state and has been used in both planar regular configurations and in inverted perovskite configurations, with the highest recorded *PCE* of 15.73% [112,113]. The first successful breakthrough for NiO_x_ as an HTL was accomplished using low-temperature solution–combustion deposition by Bai et al. (2014), who used nickel nitrate and glycine as the combustion precursors and achieved a *PCE* of 6.42% (Figure 8) [114]. In 2015, Lai and colleagues used an absorbing layer of CH_3_NH_3_PbI_3_ with NiO_x_ as an HTL, resulting in an enhanced *PCE* of 7.75% [115]. 

Various methods have been employed to improve the low efficiency of perovskite-based NiO_x_. One method is doping the metal oxide HTL to enhance the hole mobility and reduce defects. In 2015, an efficiency of 17.8% was achieved using a CH_3_NH_3_PbI_3_ active layer along with Cu-doped NiO_x_ as the HTL [116]. In 2022, Zhao et al. enhanced the *PCE* of PSCs using phthalocyanine (Pc)-doped NiO_x_, achieving a high *PCE* of 21.68% [117]. In addition to the choice of HTL material, the synthetic method and thin-film deposition route are crucial factors that contribute to the improvement of device performance. To date, the sol–gel method is considered the most appropriate technique for depositing a thin layer of NiO_x_ onto an ITO substrate in an inverted configuration.

#### 4.2.2. Nickel Cobaltite (NiCo_2_O_4_)

Nickel cobaltite (NiCo_2_O_4_) is a transparent semiconductor oxide material that has a cubic spinel crystal structure with all of the Ni ions residing on octahedral sites, while the Co ions occupy both the tetrahedral and octahedral sites [118]. Both Ni and Co are readily available transition metal elements. Therefore, various synthesis methods, including hydrothermal and solvothermal [119], combustion [120], sol–gel [121], liquid-phase co-precipitation [122], and microwave-assisted methods [123], have been utilized to synthesize NiCo_2_O_4_ at many scales. NiCo_2_O_4_, which has a bandgap of approximately 2.2–2.4 eV and a valance of 5.3 eV, has been widely used as a p-type semiconductor for storing charge in electrode supercapacitors [124,125], anodic evolution [126], organic and inorganic electro-synthesis [127], and as a substitute for the iodic electrolyte in P-type DSSCs for optoelectronic devices [128]. To the best of our knowledge, NiCo_2_O_4_ has only been used as a CTL in perovskites since 2018 due to its intrinsic properties that complement the widely used light-absorbing CH_3_NH_3_PbX_3_. 

**Figure 9:**
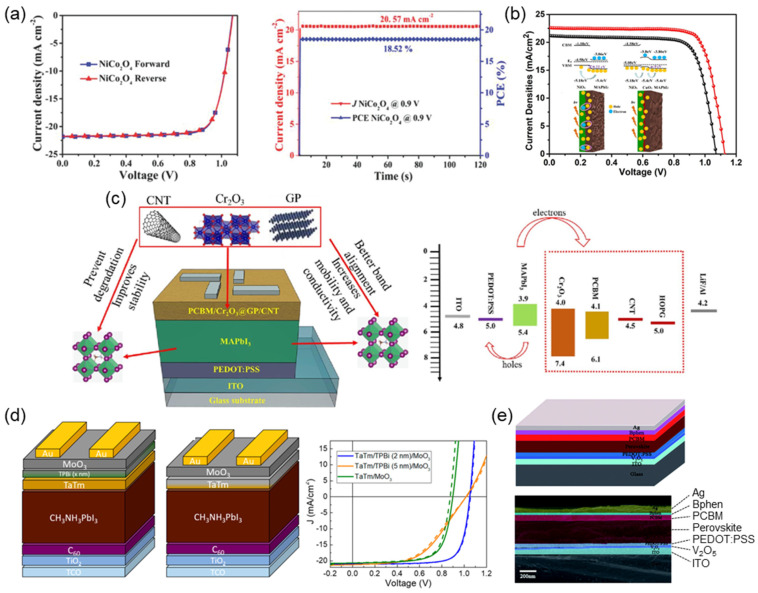
(**a**) Current–voltage (J-V) characteristics and steady photocurrent characteristics of PSCs based on NiCo_2_O_4_ tested under AM 1.5 G illumination with an intensity of 100 mW cm^−2^. (**b**) p-i-n and n-i-p configurations for use of metal oxides as CTLs for both electrons and holes, leading to a higher *PCE*. (**c**) Inverted PSCs with a *PCE* of 19.91%, 14.6% higher than the control device, demonstrating promise of interfacial layer carrier transport for high-performance PSCs and expanding PSC material options. (**d**) Schematic and cross-sectional FESEM image of a device containing V_2_O_5_. (**e**) Hybrid ETLs with Cr_2_O_3_@GP and Cr_2_O_3_@CNT with an improved *PCE* of 18.5% and 26.8% compared to a plane ETL. (**a**) Reproduced with permission [129], Copyright (2018) by John Wiley and Sons. (**b**) Reproduced with permission [130], Copyright (2021) by Elsevier’s Group. (**c**) Reproduced with permission [131], Copyright (2023) by Elsevier’s Group. (**d**) Reproduced with permission [132], Copyright (2019) by American Chemical Society. (**e**) Reprinted [133], Copyright (2017) by Royal Society of Chemistry.

Ultra-small nanoparticles (NPs) of NiCo_2_O_4_ were synthesized by Ouyang et al. in 2018 by controlling the deamination of Co-NH_3_ Werner-type coordination compounds and employed as an HTL with an efficiency of 18.23%. Their work described a new approach for the controlled deamination of Co-NH_3_ complexes in a system that included Ni(OH)_2_, resulting in the synthesis of very small NiCo_2_O_4_ NPs with an average size of 5 nm. These NPs were well-dispersed and did not require any unusual ligands. As a result, it was possible to create films that were uniform and devoid of any pinholes. NiCo_2_O_4_ films promoted the growth of large perovskite grains and minimized film imperfections. Using NiCo_2_O_4_ NPs as the HTL, the PSC achieves a notable *PCE* of 18.23% and demonstrates a favorable stability by maintaining 90% of its *PCE* after 500 h of light exposure (Figure 9a). Also in 2018, NiCo_2_O_4_ was prepared using a low-temperature combustion method resulting in a *PCE* of 15.5%. In addition, the highest *PCE* achieved to date for NiCo_2_O_4_ was achieved by doping NiCo_2_O_4_ with magnesium (Mg) in an inverted configuration, resulting in a *PCE* of 16.71%.

Other transition metal oxides, such as CuO_x_, CrO_x_, MoO_3_, V_2_O_5_, and WO_3_, are widely used in various industries, such as optoelectronics and photovoltaics, due to their excellent conductivity, transparency, high charge mobility, and abundance. Despite their n-type characteristics, these transition metal oxides are being increasingly being used as HTLs due to their high efficiency, stability, large band gap, high work function, and good hole transport properties. Interestingly, before being used as HTL materials, these transition metal elements were used as doping agents to enhance the charge mobility of organic HTLs.

#### 4.2.3. Copper Oxide (CuO_x_)

CuO_x_ has a monoclinic crystal structure and optical, electrical, and magnetic properties that make it suitable for various battery, supercapacitor, near-infrared, and magnetic storage applications. In PSCs, CuO_x_ was used for the first time as an HTL. It was prepared using a facile solution-processing method and was used in an inverted planar heterojunction PSC device with methylammonium lead halide as the active perovskite layer. The device demonstrated a *V_oc_* of 0.99 V, a *J_sc_* of 23.2 mA cm^−2^, an *FF* of 74.4%, and a total *PCE* of 17.1%. A straightforward and efficient technique has also been devised to improve the movement of carriers using a layer of CuO_x_ QDs at the interface in inverted PSCs (Figure 9b). The enhanced mobility of CuO_x_ QDs in the interfacial layer significantly improved the performance of the PSC by facilitating the more efficient transfer of electrical carriers. The enhanced crystallinity of the perovskite layer on the CuO_x_ QDs layer reduced the number of charge trap states, increasing the resistance to carrier recombination. Consequently, the inverted PSCs demonstrated a *PCE* of 19.91%, which was 14.6% higher than the *PCE* of a control device.

#### 4.2.4. Chromium Oxide (CrO_x_)

CrO_x_ is well-known for its different oxidation states, including Cr^3+^, Cr^4+^, and Cr^6+^ at 200 °C, which exhibit complex amorphous traits on a small scale. Despite being an n-type transition metal, the application of CrO_x_ as an HTL relies on the multivalence state of the metal cation. CrO_x_ was utilized for other purposes before PSCs, such as being employed as an HTL in organic solar cells via radiofrequency sputtering on fluorine-doped tin oxide (FTO). Cr_2_O_3_ has superior stability when exposed to UV radiation and has a more suitable band alignment with the perovskite layer, resulting in advantageous electron extraction. The subpar conductivity and mobility of Cr_2_O_3_ can be enhanced by the production of hybrid composites incorporating graphitic particles (GPs) and carbon nanotubes (CNTs). Hybrid ETLs consisting of Cr_2_O_3_@GP and Cr_2_O_3_@CNT have demonstrated an enhanced *PCE* of 18.5% and 26.8%, respectively, compared with a plain ETL. With the addition of Cr_2_O_3_@GP/CNT to the ETL, there was a notable improvement in the conductivity of the ETL, leading to a decrease in the series resistance (Figure 9c). As an amorphous material, CrO has received less interest from researchers. It is seldom used as a hole carrier in PSCs in its pristine state, but it is often utilized as a doping agent for other HTLs.

#### 4.2.5. Molybdenum Oxide (MoO_3_)

Despite being an n-type semiconductor, molybdenum oxide (MoO_3_) is utilized in PSCs as an HTL. MoO_3_, which crystallizes in an orthorhombic crystal structure and has different polymorphs, has a high work function, which allows it to readily extract and transport holes that form in the perovskite layer. This makes it easier for these holes to travel to the electrode. MoO_3_ improves the long-term performance of PSCs because of its stability and compatibility with other cell components. It is possible to exercise fine control over the thickness of the MoO_3_ layer, which enables the performance of the device to be optimized. As shown in Figure 9d, MoO_3_ has been used to improve carrier extraction to the transparent bottom electrode in a p-i-n configuration. It can also be used on top of a perovskite absorber in an n-i-p configuration by inserting a molecular interlayer with a lone pair nitrogen atom and a low ionization potential. The first vacuum-deposited PSCs containing metal oxides as CTLs for electrons and holes were made possible by this method, resulting in a *PCE* of over 19%. MoO_3_ has been extensively used as a hole-injection or hole-extraction layer in organic-LEDs, as well as in organic and QD solar cells.

#### 4.2.6. Vanadium Peroxide (V_2_O_5_)

V_2_O_5_ is the most stable form of vanadium and has a diverse range of applications, from catalysis to optoelectronic devices. It can be synthesized through various methods, including solution processing and vapor deposition. It is used as a counter electrode in DSSCs and as a hole carrier material in inverted PSCs and organic solar cells despite its n-type state because of its resistance to harsh weather conditions.

A high-performance device was created using a V_2_O_5_ film to alter a PEDOT:PSS HTL, based on a standard PSC. The addition of a V_2_O_5_ layer prevents the corrosive effect of the acidic PEDOT:PSS film on the ITO electrode, thus improving the durability of the PSC structure (Figure 9e). The V_2_O_5_ film exploits the distinct refractive index properties of the neighboring layers to enhance the absorption of photons by the perovskite layer, resulting in a higher *PCE*.

### 4.3. Small Molecule-Based HTMs 

Traditional HTLs have been commonly used in PSCs for their efficiency and stability. However, they have notable limitations, including high costs, complex synthesis processes, and limited long-term durability. Therefore, research has increasingly turned toward innovative small molecule-based HTMs, which offer greater tunability, cost efficiency, and, often, improved performance, marking a significant shift in PSC technology.

#### 4.3.1. Sulfonated HTMs 

One promising avenue in small molecule-based HTMs is the development of sulfonated materials. Sulfonation introduces sulfonic acid groups into the HTM structure, which can improve solubility and conductivity. These groups also enhance the material’s ability to form strong intermolecular interactions, thereby improving film quality and stability. For instance, sulfonated benzothiadiazole derivatives have shown excellent performance in PSCs, with enhanced stability and charge mobility compared to conventional HTMs. Such materials enable more efficient hole extraction and transport due to improved film morphology and reduced interface resistance. Sulfonation has also been explored for enhancing hydrophilicity, which in turn leads to improved compatibility with the perovskite layer [134].

#### 4.3.2. Hydrogen-Bonding HTMs

Another approach involves introducing hydrogen-bonding functionalities into HTMs. Hydrogen bonds can help maintain structural integrity, improve molecular packing, and enhance hole mobility by reducing the energetic disorder within the HTL. For instance, small molecules with amine or hydroxyl groups capable of forming hydrogen bonds exhibit improved thermal and mechanical stability in PSC devices [135,136]. By promoting strong molecular interactions, these HTMs minimize voids and enhance charge transport properties. Hydrogen-bonded HTMs, such as carbazole derivatives with NH groups, have demonstrated improved stability and hole mobility, as well as better performance under operational conditions [137,138].

#### 4.3.3. Halogen-Bonded HTMs 

Halogen bonding is another strategy that has recently attracted interest in improving HTM performance. Halogen bonds, like hydrogen bonds, provide non-covalent interactions that enhance molecular packing and stability. Halogen bonding can lead to improved crystalline ordering in HTL films, enhancing charge mobility and reducing recombination losses. Small molecules containing halogen groups, such as iodine-substituted triphenylamine derivatives, have shown promising results, offering stability and performance comparable to or better than traditional HTMs in PSCs. Furthermore, halogen bonding has been associated with enhanced ambient stability due to better film quality and hydrophobicity, which helps protect the perovskite layer from moisture [139].

#### 4.3.4. π-Conjugated Small Molecules

π-Conjugated small molecules, such as diketopyrrolopyrrole and thiophene derivatives, also represent a growing field in HTM development for PSCs. These molecules offer strong π–π stacking interactions that enhance charge mobility, ensuring efficient hole transport. Their conjugated structure and customizable side groups allow for bandgap tuning, enabling compatibility with different perovskite compositions. Additionally, these materials are often synthetically simpler and less costly to produce than Spiro-OMeTAD, making them attractive for large-scale applications. Recent studies show that π-conjugated small molecules can achieve performance levels on par with or superior to PTAA and Spiro-OMeTAD, with improved stability and cost efficiency [140].

#### 4.3.5. Functionalized Triphenylamines 

Functionalized triphenylamines, particularly with electron-donating groups, are another type of small molecule HTM gaining attention. Their structure allows for customization to optimize energy levels for hole injections, improving the efficiency of hole transport. Some triphenylamine derivatives exhibit excellent environmental stability and high hole mobility. Functionalization with groups such as methoxy or dimethylamino further tunes the energy levels and increases the HTM’s hydrophobicity, enhancing its compatibility with perovskite layers and improving stability under ambient conditions [141].

## 5. Electron Transport Layers (ETLs)

ETLs, which are n-type semiconductors, have historically been used for electron storage or conduction. In PSCs, these materials serve as layers where electrons are captured after being extracted from the active material during the excitation and separation mechanisms within the active layer. The ETL then directs the electrons to the transparent conductive oxide (TCO), usually ITO or FTO. During charge separation, the ETL has two roles: to prevent holes from entering the counter electrode and to transport electrons to the appropriate electrode. The use of an ETL in the PSC increases the stability and efficiency and offers almost defect-proof performance depending on the device configuration [142]. An ideal ETL for a PSC should be spreadable when deposited onto the designated layer and have high optical transmittance. Additionally, the energy band alignment between the energy levels of the device must be suitable. The ETL should also be resistant to high temperatures and moisture, and affordable synthetic routes should be available. The materials used should follow previously established principles to ensure continuity in the development process. Various materials, particularly n-type semiconductors such as metal-oxide-based transition compounds and carbon-based molecules have been synthesized, characterized, and extensively tested as ETLs.

### 5.1. Organic ETLs: Fullerene-Based Materials

**Figure 10:**
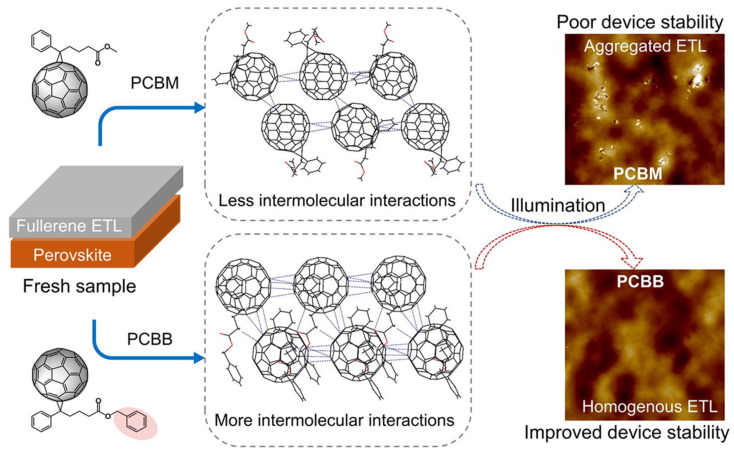
Use of fullerenes in PSCs as an ETL. Reproduced with permission [148], Copyright (2021) by Elsevier’s Group.

Fullerene and its derivatives, including PC_61_BM, PC_71_BM, and indene-C60 bis-adduct (ICBA), are carbon-based materials that have a wide range of applications in various industrial fields. As shown in Figure 10, these materials were first introduced in organic photovoltaics and perovskite photovoltaics as ETL materials, particularly in inverted perovskite devices [143,144]. Fullerene derivatives have gained significant attention due to their ability to address some of the limitations of other ETLs. They are effective hole-blocking materials, passivating charge traps at the interface of the light-absorbing layer and promoting efficient charge transfer to the electrode, which helps to reduce device hysteresis [145,146]. Of these materials, [6,6]-phenyl-C61-butyric acid methyl ester (PCBM) is the most widely used organic n-type semiconductor as an electron acceptor in PSC devices. PCBM has a HOMO energy level ranging from –5.8 to –6.0 eV, a LUMO estimated to be between –3.7 and –4.0 eV, and an electron mobility of around 10^−2^ cm^2^ V^−1^ S^−1^ [147]. In the past, PCBM was widely used as an active layer with p-type organic semiconductor compounds to generate charge carriers for photovoltaic applications. ETLs that are based on fullerenes exhibit favorable defect passivation and enhance the performance of inverted PSCs (Figure 10). Nevertheless, the π-cage structures of fullerenes result in self-aggregation, which reduces the stability of the corresponding polystyrene compounds over an extended period.

In 2013, PCBM was first used as an ETL in an inverted PSC design. The exceptional characteristics of fullerenes, including their high electron affinity, superior electron mobility, and energy levels that align well with perovskite materials, were effectively demonstrated in this application. Fullerene derivatives can also passivate the trap states found on the surface and grain boundaries of perovskite layers. This passivation process effectively reduces the unwanted hysteresis and enhances the *PCE* of PSCs. A set of fullerene derivatives, modified with cross-linkable groups such as ester, amino, and pyridine groups, have thus been developed and employed as ETLs in inverted PSCs with the aim of improving electron extraction and removing defects at the interface between the fullerene and perovskite, ultimately leading to higher *PCE*s.

Demonstrating the impact of the molecular structure of the modified fullerene on aggregation, a variant of [6,6]-phenyl-C61-butyric acid benzyl ester (PCBB) was created by adding an additional phenyl group. This modified PCBB was then integrated into an inverted PSC as an ETL. Analysis of the crystal structure revealed that PCBB had considerably higher intermolecular contact, such as π–π interactions between the C60 cages and CH–π interactions between functional groups, in comparison to PCBM. These interactions may inhibit the formation of aggregates in fullerene-based ETLs. Consequently, a device equipped with PCBB as an ETL produced a *PCE* of 19.84%. PCBB-based devices also demonstrated superior long-term stability at the maximum power point over a period of 600 h, surpassing the stability of PCBM-based devices. ICBA, another fullerene-based compound, is occasionally used as an ETL, with a slightly higher unoccupied molecular orbital than PCBM and higher solubility in DMF. ICBA is a competitive organic electron carrier candidate because of its ability to suppress defects that undermine the performance of solar cells [148].

### 5.2. Inorganic ETLs

#### 5.2.1. Titanium-Based ETLs

**Figure 11:**
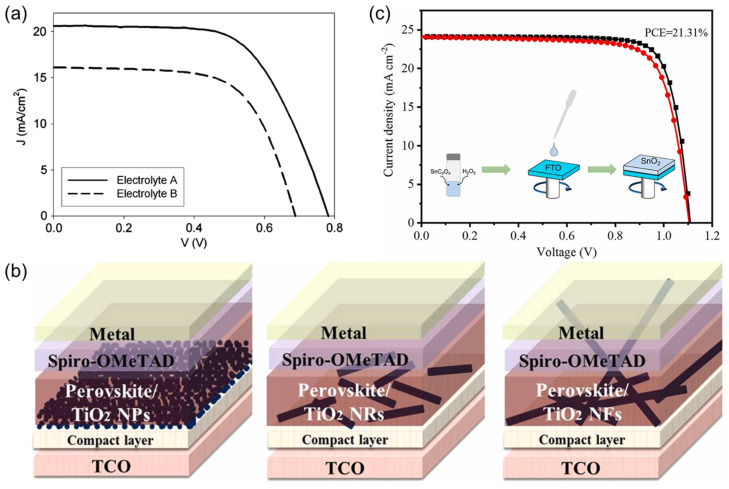
(**a**) Relationship between current and voltage in a DSSC with acetonitrile and an ionic liquid as electrolyte. (**b**) Structure of an FTO/TiO_2_ compact layer/TiO_2_ NS layer/perovskite/Spiro-OMeTAD/Au device used for the fabrication of PSCs in ambient conditions. (**c**) High levels of SnC_2_O_4_ were used to produce SnO_2_ films with an improved *PCE* of 21.31%. (**a**) Reproduced with permission [149], Copyright (2009) by Elsevier’s Group. (**b**) Reproduced with permission [150], Copyright (2023) by Elsevier’s Group. (**c**) Reproduced with permission [152], Copyright (2022) by Elsevier’s Group.

In 1791, William Gregor discovered TiO_2_ in black magnetic sand in Cornwall. Commercialization of this naturally occurring oxide began in the 1920s. TiO_2_ exists in four different phases, including two phases with a tetragonal crystal structure (anatase and rutile), one phase with an orthorhombic structure (brookite), and TiO_2_-B with a monoclinic crystal structure. TiO_2_ has various applications in different fields, such as pigmentation, sunscreens, paints, ointments, and toothpaste. The production of pigmentation involves reacting ilmenite (FeTiOa) with sulfuric acid, followed by a hydrolysis procedure that incorporates Ca or barium sulfate. As shown in Figure 11a, TiO_2_ paste has been successfully synthesized using a Pechini sol–gel method and nanocrystalline TiO_2_ powder. TiO_2_ layers have a high inner surface area, which absorbs high quantities of dye, and well-connected nanocrystalline grains, which allow electrons to move freely within the layer. The dye-sensitized layers were used in DSSCs with an electrolyte consisting of acetonitrile and ionic liquid. The overall conversion rate for the DSSCs was 10.2% with acetonitrile and 7.3% with an ionic liquid-based electrolyte at 100 mW cm^−2^, 25 °C, and AM 1.5 G [149]. 

Multiple metal oxide semiconductor materials have been utilized as electron acceptors in PSCs, but TiO_2_ remains the most extensively studied and utilized due to its exceptional properties. With a band gap of approximately 3.0–3.2 eV and light absorption of about 400 nm, TiO_2_ has high transmittance, which assists in suppressing photo-resistance. It is also cost-effective and chemically stable. The use of alternative NPs to TiO_2_ as an ETL in FTO/TiO_2_ compact layer/TiO_2_ NS layer/perovskite/Spiro-OMeTAD/Au solar cells have also been explored (Figure 11b). The findings suggest that the use of TiO_2_ nanorods as an ETL provides a direct pathway for electrons and produces an acceptable charge recombination resistance, resulting in a remarkable efficiency of 13.87% [150,151]. A consistent layer of anatase-dominated TiO_2_ was deposited on mesoporous TiO_2_, leading to a decrease in the defect density and an improvement in the band energy level. The suppression of interfacial charge recombination led to a concomitant improvement in the charge extraction efficiency of the solar cells. The *PCE* of carbon electrode-based PSCs (C-PSCs) was enhanced from 16.63% to 18.08%, representing the highest efficiency achieved for a C-PSC using a wide-bandgap perovskite [151]. Despite its low electrical conductivity, TiO_2_ remains the most efficient metal oxide ETL and is commonly used as a compact and mesoporous material in planar n-i-p perovskite configurations.

#### 5.2.2. Tin-Based ETLs

SnO_2_ can be used as a direct substitute for TiO_2_ to extract and transport electrons in PSCs. Initially, SnO_2_ was used as a compact and mesoporous ETL in a planar configuration with an efficiency of approximately 17%. This material has more suitable properties than TiO_2_ for photovoltaic applications, including a suitable band alignment with perovskites, more favorable optical properties, a wide band gap that is highly resistant to UV radiation, and outstanding electrical properties. In addition, SnO_2_ can be prepared easily and inexpensively using various solution methods, making it a desirable low-cost alternative with high stability and a long lifetime. The reported efficiency of PSCs with SnO_2_ as the ETL has been impressive, with the possibility of tuning the SnO_2_ properties during thin film deposition. Cheng et al. prepared SnO_2_ using a technique designed for the rapid, clean, and energy-efficient fabrication of SnO_2_ ETLs with a precursor solution of tin oxalate (SnC_2_O_4_) in hydrogen peroxide at a temperature of 180 °C (Figure 11c). When the SnC_2_O_4_ concentration was optimized, high-quality SnO_2_ films were produced. These films improved the long-term stability of the PSC and led to an improved *PCE* when compared to reference solar cells made with commercially available SnO_2_ films. In particular, the efficiency of the reference solar cell was 20.40%, while the target solar cell produced a *PCE* of 21.31%, the highest reported to date for this metal oxide as an ETL. It is important to note that the doping of pure SnO_2_ is necessary to improve the ETL quality in situations involving lattice mismatch or interfacial defects resulting from the interposition of different perovskite layers [152].

#### 5.2.3. Zinc-Based ETLs

Zinc oxide (ZnO) is a semiconductor metal oxide that functions as a charge acceptor in electronics and optoelectronic devices. Due to its excellent properties, such as a high electron mobility, high thermal conductivity, and large exciton binding energy, ZnO has been widely studied for its potential applications in transparent thin-film transistors, photodetectors, LEDs, laser diodes, and solar cells [153,154]. For example, ZnO was employed in the inverted organic solar cell developed by Assaf Manor et al., which demonstrated improved electrical characteristics, while Savva and co-workers used ZnO to dope Cs as a hole-blocking layer in an inverted organic solar cell, resulting in improved device performance [155,156]. ZnO is useful as a hole-blocking layer in PSCs because it has similar properties to TiO_2_ and SnO_2_ along with superior electron mobility and an affinity that matches the perovskite energy level. However, pure ZnO can lead to defects in the perovskite active layer, such as the formation of zinc hydroxide, which can be overcome by modifying the material. Zinc sulfite (Zn_2_SO_4_) and zinc selenide (ZnSe) have been proposed as alternatives to extract electrons and prevent the passage of holes in PSCs. These chalcogenide semiconductors, derived from the alkali metal and chalcogen elements, are in the early stages of development and have the potential to improve the performance of perovskite devices. In addition, by adding Cs or Li dopants to the ZnO layer and then depositing a self-assembled monolayer on top, its properties can be altered. By manipulating the bulk and surface properties of ZnO simultaneously, planar MAPbI_3_ PSCs can achieve a maximum *PCE* of 18%, while also reducing hysteresis and greatly improving the stability of the device [157]. 

**Figure 12:**
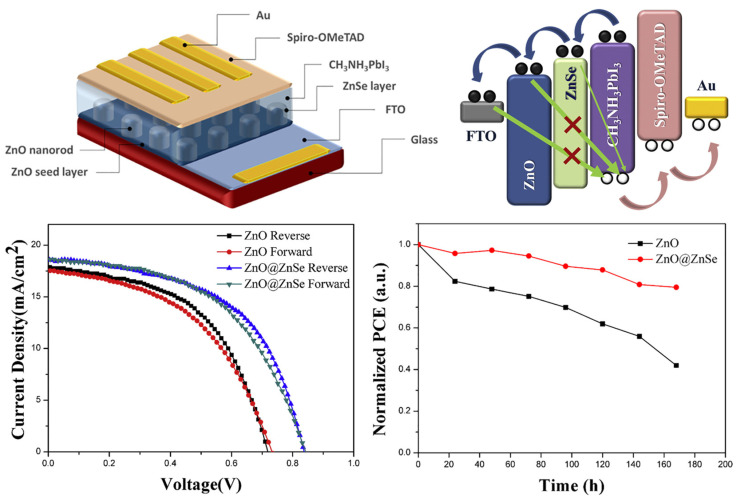
*PCE* of a ZnSe-modified PSC. Reproduced with permission [159], Copyright (2019) by Elsevier’s Group.

Nevertheless, it is important to resolve the instability of the perovskite absorber layer deposited on ZnO, which leads to a low *PCE* and long-term stability issues, in order to fully take advantage of ZnO as the ETL in PSCs. To address this, a previous study applied a spin-coating technique to deposit an ultrathin layer of niobium pentoxide (Nb_2_O_5_) on the surface of ZnO to act as a surface passivation layer. Both ZnO and Nb_2_O_5_ were produced by spin coating, then sintered at a relatively low temperature of 200 °C. By employing Nb_2_O_5_ surface passivation and low-temperature-processed ZnO as the ETL, the stability of the perovskite film was significantly improved over a period of 20 days under normal environmental conditions [158]. Another study investigated the impact of ZnSe on the morphology, structure, light absorption, and charge transfer of perovskite films (Figure 12). It also examined the performance of PSCs based on ZnO nanorod arrays. The *PCE* of the PSC was greatly increased by 27% with the addition of ZnSe. This improvement may be ascribed to the appropriate alignment of the energy bands, improved separation of charge carriers, and the efficiency of charge transfer. The use of ZnSe also enhanced the air stability of the PSCs [159].

#### 5.2.4. Other Inorganic ETL Materials

The use of precisely designed binary and ternary metal oxide semiconductors as buffer layers has become widespread due to their excellent energy-level matching with the perovskite active layer and a performance and stability that are comparable to those of commonly used buffer layers such as TiO_2_, SnO_2_, and ZnO [160]. Of the n-type metal oxides used as buffer layers, cerium oxide (CeO_2_) is a notable addition. Researchers have combined CeO_2_ with PCBM to form an ETL for bilayer structures and achieved a remarkable *PCE* of 18.7% [161]. A Pb-free perovskite device with a *PCE* of 17.77% has also been analyzed using SCAPS 1D simulation software [162]. Another potential candidate for use in PSCs is Nb_2_O_5_, which can block holes and extract negative charge to the cathode. Its bandgap can be easily adjusted, and it exhibits high stability, making it a promising option for photovoltaic applications [163]. Lead-based perovskites, particularly methylammonium lead iodide, have demonstrated exceptional power conversion efficiencies. However, the toxicity associated with lead poses serious environmental and health risks, raising questions about the long-term sustainability of lead-containing PSCs. This concern has catalyzed research into lead-free perovskite materials that aim to retain the advantageous optoelectronic properties of traditional perovskites while eliminating or significantly reducing the use of toxic elements. The primary focus has shifted to materials that incorporate elements such as tin (Sn), bismuth (Bi), and germanium (Ge) as potential replacements for lead. Among these, tin-based perovskites are of particular interest due to their relatively high photoconductivity and suitable bandgap for solar energy applications. However, tin perovskites face challenges such as rapid oxidation and instability in ambient conditions, which limit their practical application. To mitigate these issues, recent advancements have explored mixed cation and halide compositions to stabilize tin-based perovskites. For example, incorporating cesium and formamidinium can enhance the thermal and phase stability of tin perovskites, allowing for improved performance in PSCs [164]. Additionally, the development of novel synthetic routes, such as the use of additive engineering and encapsulation techniques, has shown promise in addressing the inherent instability of tin-based materials. 

Another noteworthy lead-free alternative is bismuth-based perovskites and other related compositions, have garnered attention due to their non-toxic nature and potential for use in optoelectronic devices. These materials exhibit unique properties, such as a direct bandgap and favorable charge transport characteristics, making them suitable candidates for PSCs. Although the power conversion efficiencies of bismuth-based perovskites currently lag behind lead-based systems, ongoing research is dedicated to improving their performance. Techniques such as doping with other elements and optimizing the material’s morphology have been investigated to enhance efficiency and stability [165]. The sustainability of lead-free perovskites extends beyond their non-toxic composition; it also encompasses their lifecycle from synthesis to disposal. Recent life cycle assessments of lead-free PSCs indicate that these materials could offer a more environmentally friendly alternative to traditional lead-based systems, provided that efficient recycling methods and waste management strategies are developed [166]. Innovations in fabrication processes, such as low-temperature solution processing, can further reduce energy consumption and enhance the sustainability of PSC production. Additionally, integrating lead-free perovskites into existing solar cell technologies can foster a transition toward greener energy solutions. The successful implementation of lead-free PSCs not only addresses health and environmental concerns but also aligns with the growing global emphasis on sustainability and renewable energy sources. In summary, lead-free perovskites represent a crucial area of research that addresses the pressing toxicity concerns associated with lead-based materials. While challenges remain in achieving efficiencies comparable to their lead-containing counterparts, significant advancements are being made in developing stable, high-performance lead-free alternatives. By incorporating these materials into our discussion, we will contribute to a broader understanding of the future of sustainable solar energy technologies.

## 6. Electrodes and Counter Electrodes for PSCs

**Figure 13:**
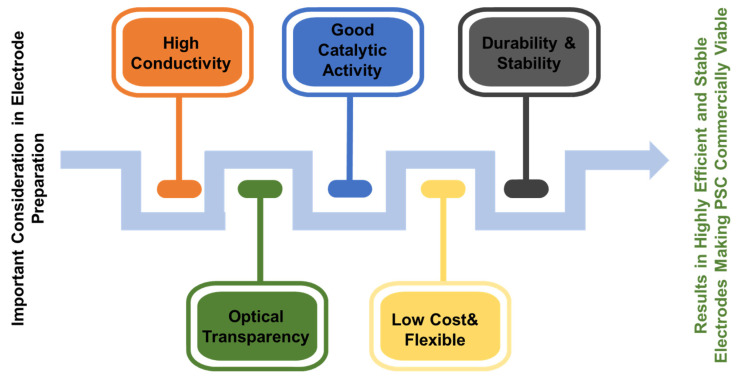
Development of counter electrodes for more efficient, stable, and financially feasible PSCs for solar energy market adoption.

The extremely low production costs and excellent efficiency of PSCs have positioned them as a next-generation photovoltaic technology. However, optimizing the operation and performance of PSCs requires a clear understanding of the roles of the transparent conductive electrode (TCE) and counter electrode (Figure 13). The TCE has several functions, one of which is to act as the anode in a PSC. The perovskite layer absorbs photons and generates charge carriers via a transparent substrate that allows incoming light to pass through. Two materials that are often used for the TCE are ITO and FTO. However, ITO is limited by its high material and processing costs, its high brittleness, and the scarcity of indium. To improve the performance and longevity of PSCs, researchers are investigating new materials for use in electrodes. In addition to absorbing light, the TCE is an essential point of contact for collecting holes produced by light. Under light illumination, the perovskite layer generates electron–hole pairs. As they move toward the TCE, the holes generate an electric current in the external circuit. For highly efficient PSCs, the TCE needs excellent charge extraction capabilities, transparency, and high conductivity.

On the other side of the perovskite layer, the counter electrode acts as the cathode for the PSC. To complete the electrical circuit, the counter electrode collects electrons produced by the perovskite layer. To achieve effective charge extraction, avoid recombination losses, and ensure the long-term stability of the PSC, the choice of the counter electrode material is crucial. The high conductivity of metals such as Au and silver (Ag) makes them popular choices for the counter electrode, while conductive polymers, such as PEDOT:PSS, have also been employed because they offer several benefits, including being easy to produce, inexpensive, and flexible. However, although they are strong conductors, the high price and limited availability of Au and Ag remain important limitations, while conductive polymers may lack interface compatibility and long-term stability. 

To optimize performance, affordability, and sustainability, and thus realize the full potential of PSCs as an efficient and environmentally friendly renewable energy source, researchers have devoted significant resources to studying innovative electrode designs and material combinations [58,167]. For example, a novel approach that has been proposed is the creation of hybrid electrodes that combine several materials to maximize their respective strengths. These electrodes can include carbon-based structures, metal oxides, and conductive polymers. In addition, new deposition methods have been investigated to improve the stability and performance of PSCs by fabricating electrodes with more precision and control. These methods include chemical vapor deposition and atomic layer deposition. It is also important to use electrode materials and production processes that are both scalable and inexpensive to allow for commercialization.

## 7. Interface Engineering in PSCs

### 7.1. Perovskite Interface with ETLs and HTLs

**Figure 14:**
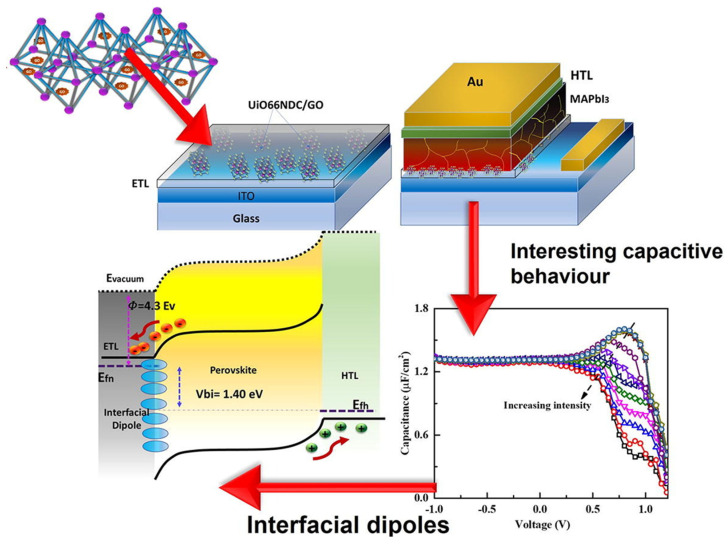
Delocalized surface states in PSCs. Reproduced with permission [168], Copyright (2019) by Elsevier’s Group.

The interfaces between the perovskite layer and the ETL and HTL strongly affect the efficiency of PSCs. Efficient charge transfer and extraction are crucial in these devices to optimize energy conversion. Electron transport often uses materials such as TiO_2_ and ZnO to assist in the retrieval and transport of electrons produced by the perovskite layer when it absorbs sunlight. Similarly, effective hole transport depends on the use of appropriate materials, with Spiro-OMeTAD being particularly notable. The relationship between perovskite and these transport materials is complex. Defects or traps present at the interface can result in charge recombination, reducing the overall efficiency of the solar cell. Researchers have thus concentrated on optimizing these interfaces to increase the movement of the charge carriers, minimize losses due to recombination, and enhance the overall stability of the device. As shown in Figure 14, interface engineering is key to improving charge carrier extraction in metal–halide PSCs. For example, the charge transfer of a tin oxide ETL was improved by adding a metal–organic framework to the perovskite absorber layer–ETL interface. This reduced the number of trap states to improve the growth of the perovskite film. This led to a ~12% increase in device efficiency. The capacitive behavior was also studied to understand charge buildup and transfer [168]. As this study demonstrated, it is essential to customize the characteristics of ETLs and HTLs and to refine their application techniques to obtain highly efficient PSCs as a sustainable and economically viable solar energy option.

### 7.2. Surface Passivation Techniques

Surface passivation is used to improve both the performance and stability of PSCs by removing flaws, improving the charge carrier lifetime, and limiting non-radiative recombination. This involves depositing a thin layer of an organic or inorganic material on the surface of the perovskite. Due to this passivation, ion migration is prevented, trap states can be reduced, and the overall electronic characteristics of the perovskite layer can be improved significantly. This approach thus improves *V_oc_*, reduces hysteresis, and improves long-term stability.

### 7.3. Interface Characterization Methods 

Characterizing the interfaces in PSCs is required to optimize device performance. Various methods are employed to analyze the interactions between the perovskite layer, ETL, and HTL. Techniques such as X-ray photoelectron spectroscopy provide insights into chemical composition, helping to detect the presence of defects or contaminants. Kelvin probe force microscopy assesses variation in the surface potential and aids in the understanding of the charge carrier dynamics. Time-of-flight secondary ion mass spectrometry maps elemental distributions at the interfaces, thus informing the choice of materials. Additionally, impedance spectroscopy measures charge transport and recombination kinetics. Combining these methods allows for a comprehensive understanding of interface properties, enabling researchers to refine material choices, deposition processes, and passivation strategies for improved PSC efficiency and stability.

In PSCs, generally, interfacial engineering plays a pivotal role in reducing charge recombination, enhancing charge extraction, and improving overall stability. Interfaces, particularly those between the perovskite absorber and adjacent HTL/ETL, are often sites for defect accumulation and energy mismatches. These interfacial defects can trap charge carriers, leading to non-radiative recombination, thereby lowering the *PCE* and affecting stability. Furthermore, mismatches in energy levels between the layers result in inefficient charge transfer, limiting device performance. One of the most promising approaches in interfacial engineering is defect passivation, which involves the reduction or neutralization of interfacial trap states. A range of materials has been employed for this purpose, including self-assembled monolayers, which modify surface energy and reduce trap density, and two-dimensional (2D) materials, such as graphene and transition metal dichalcogenides, which can act as barrier layers that enhance charge extraction. Self-assembled monolayers are particularly useful in providing a tailored surface that minimizes non-radiative recombination and promotes better alignment of energy levels. They create a more favorable interface for charge transfer, leading to reduced interface resistance and improved *PCE*. Meanwhile, 2D materials not only improve charge transport but also introduce hydrophobicity, which can shield the perovskite layer from moisture, a primary cause of degradation [169]. Furthermore, recent studies have introduced functionalized small molecules, such as fullerene derivatives and alkylammonium salts, as interfacial modifiers. These materials enhance the PSC’s stability by suppressing ion migration, a known contributor to device degradation. Fullerene-based compounds, in particular, have shown promise in stabilizing the perovskite/ETL interface by passivating the ionic defects present on the perovskite surface, thereby improving both device efficiency and stability under prolonged illumination. Alkylammonium salts have also demonstrated the capability to bond strongly with the perovskite layer, thus stabilizing the interface and preventing ion diffusion across the HTL and ETL interfaces [170].

### 7.4. Stability Enhancement

Device stability remains one of the most significant challenges for PSCs, as these cells are prone to degradation under environmental stresses such as moisture, UV exposure, and thermal fluctuations. This instability is mainly due to the presence of methylammonium (MA) ions in commonly used hybrid perovskite structures, which are volatile and degrade when exposed to humidity and heat. To address this, recent advances have focused on improved encapsulation strategies and compositional engineering. Encapsulation involves creating protective barriers that prevent external agents like moisture and oxygen from reaching the perovskite layer. While traditional encapsulation methods have used glass or metal layers, recent developments favor hybrid barriers combining organic and inorganic materials. For instance, polymer-based encapsulation methods have been enhanced with inorganic coatings, such as atomic layer deposition of Al_2_O_3_ which provides a dense, impermeable layer that enhances moisture resistance and improves stability under high-humidity conditions [171]. Hybrid barriers not only create robust protective layers but also maintain flexibility, making them ideal for applications in flexible and wearable PSCs. Compositional engineering also addresses stability by replacing volatile components with more stable alternatives. For example, partially substituting methylammonium with inorganic cations such as cesium or rubidium results in perovskite structures that are more thermally stable and less prone to phase segregation under operational conditions. Cesium and rubidium ions enhance the structural integrity of the perovskite lattice, allowing it to retain the desirable black perovskite phase under heat and light exposure, thus enhancing both thermal and photostability. Finally, chemical passivation of perovskite surfaces through additives such as quaternary ammonium salts, antioxidants, and crown ethers can further mitigate degradation. These compounds act by neutralizing reactive species and defects within the perovskite material itself, preventing oxygen and moisture-induced degradation. Quaternary ammonium salts have been shown to bind effectively with the perovskite surface, passivating defects and enhancing resilience against external environmental factors, while antioxidants prevent oxidative degradation, particularly at the HTL and ETL interfaces [172].

## 8. Stability and Degradation Issues 

### 8.1. Moisture and Oxygen Sensitivity

Solar cells containing perovskite are susceptible to oxygen and moisture, which influence their stability. Moisture intrusion can severely damage the structure of perovskites, reducing performance and durability. Exposure to oxygen can also result in chemical changes in the perovskite material and influence its optoelectronic properties. The use of encapsulation techniques with materials that are resistant to moisture and enhanced device layouts have been investigated as potential solutions to these problems. Addressing the sensitivity of PSCs to oxygen and moisture will improve their resistance to real-world environmental conditions and promote their long-term reliability in renewable energy applications, thus enhancing their commercial appeal.

### 8.2. Thermal Stability 

High temperatures can negatively affect the performance and reliability of PSCs due to their influence on ion migration, crystal phase changes, and thermal breakdown. These effects reduce the efficiency, increase the hysteresis, and compromise the long-term stability of the cell. These issues have been actively addressed by researchers by developing perovskite materials that are thermally stable, improving device designs, and developing new encapsulation techniques. Enhanced thermal resilience can be achieved by using more stable substrates and engineering the composition of the perovskite layer and its interfaces. Improving the thermal stability of PSCs would allow them to be employed in a variety of climates and applications, and thus facilitate their integration into renewable energy solutions.

### 8.3. Strategies for Enhanced Stability

**Figure 15:**
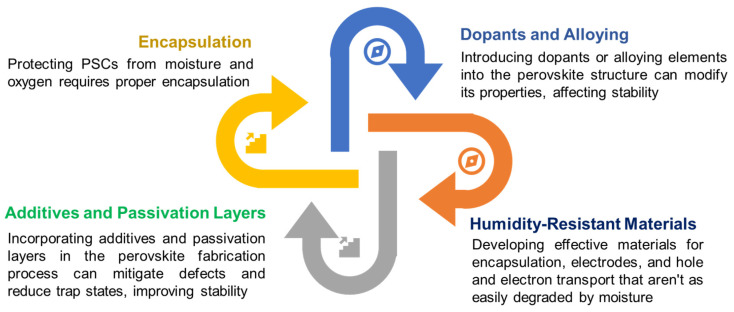
Key strategies used to enhance the stability of PSCs.

A number of approaches have been introduced to improve the stability of PSCs and reduce the number of defects, including encapsulation methods that protect against moisture and oxygen, material engineering for more stable formulations, and rigorous interface optimization to minimize defects. As shown in Figure 15, strategies that contribute to durability include the incorporation of stabilizing chemicals, the design of innovative device topologies, and the use of inert gas conditions during the fabrication process. Furthermore, efficient thermal management and rapid testing simulations can be used to identify and correct any problems in the early stages of development, contributing to the advancement of PSC technology to produce photovoltaic systems that are durable, long-lasting, and commercially viable.

### 8.4. Device Architecture for Enhanced PSC Performance

To improve the efficiency of PSCs, it is essential to optimize their architecture. Through the use of tandem structures consisting of multiple perovskite layers with variable bandgaps, it is possible to absorb a wider range of the solar spectrum, which improves the overall efficiency and increases the amount of light that is utilized. In these designs, the charge transport layer and selective contacts can reduce the resistive loss and enhance charge extraction, respectively. Increasing the amount of light that is transmitted through the front electrode using transparent conductive oxides, such as FTO or ITO, also improves light transmission. Inverted device configurations, in which the transparent conductive oxide and ETL are switched, reduce interface recombination and improve device stability. It is also possible to use flexible substrates for the development of lightweight PSCs that can be integrated with a variety of surfaces and be employed in a diverse range of applications. By incorporating these architectural innovations, higher *PCE*s, stability, and scalability can be achieved, making PSCs a more practical option for use in sustainable energy systems. The comparison of various compounds used in different layers of perovskite solar cells and their efficiencies are listed in Table 3, and a comprehensive comparative evaluation of additive components, passivation materials, transmission materials, and modified interface structures employed in perovskite solar cells are listed in Table 4. Each of these components critically impacts key performance metrics, including efficiency, stability, and device reliability, underscoring their essential role in optimizing solar cell functionality.

## 9. Future Directions and Outlook

### 9.1. Potential Applications Beyond Solar Cells

Perovskite materials can be adapted for potential use in a wide range of applications, with potential implications for technologies related to electronics, lighting, sensing, and energy storage. For example, the bandgap of perovskites can be tuned, which is advantageous for LEDs because the resulting emission spectrum is more efficient and customizable. Photodetectors based on perovskites are also highly sensitive in the visible and near-infrared spectrum, which holds great potential for the development of imaging and sensing technology. In addition, perovskites can be employed in transistors due to their excellent charge transport capabilities, which could potentially improve the performance of electronic devices. Perovskite compounds have also been shown to demonstrate potential oxygen reduction and evolution activity in catalytic applications. This suggests that perovskite compounds could be used in fuel cells and metal–air batteries. Furthermore, perovskite materials have the potential to be used in lasers because their emission wavelengths can be adjusted.

### 9.2. Integration with Other Energy Systems

**Figure 16:**
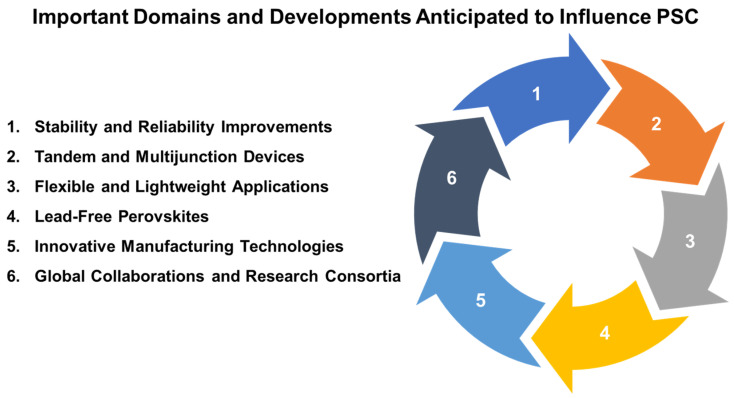
Potential directions for PSC systems.

PSCs can be incorporated into various energy systems, thus enhancing the sustainability and resilience of the energy infrastructure. An example of this involves using PSCs in hybrid energy systems that combine sustainable energy sources such as wind and hydroelectric power. This strategy takes advantage of various technologies to produce a more reliable energy delivery system with fewer interruptions. In addition, PSCs can be easily incorporated into energy storage systems, such as batteries and supercapacitors. This integration mitigates the concerns over the intermittent nature of solar energy by accumulating surplus energy produced during peak sunshine hours and using it during periods of low sunlight or increased demand. PSCs can also play a role in decentralized energy production via smart grids. Because PSCs have the potential to be integrated with building elements such as windows and facades, building structures can be transformed into sources of energy generation. In addition, the integration of tandem structures, which combine PSCs with conventional Si-based solar cells, improves the overall energy harvesting efficiency. The integration is essential for optimizing energy generation and fulfilling the requirements of an expanding and ever-changing energy environment. Ongoing research is dedicated to enhancing these integration methodologies by optimizing their efficiency, stability, and scalability (Figure 16), with the ultimate objective of producing PSCs that are essential components of a sustainable and interconnected energy future.

### 9.3. Regulatory and Policy Considerations 

Regulatory and legislative decisions impact the energy environment and can affect the broad use of PSC technology. In particular, ethical, environmentally friendly, and safe perovskite material production is greatly aided by well-established regulatory frameworks. To reduce the environmental impact of perovskite-based electronics, recycling and disposal guidelines have been established by environmental bodies. The creation and use of PSCs are also heavily influenced by incentives and laws put in place by the government. In particular, investment in perovskite technology is encouraged by financial incentives, tax credits, and research funding, which in turn fosters innovation and commercialization. To ensure responsible development, however, policies must address concerns about the toxicity, stability, and long-term environmental effects of the materials used in PSCs. Consistent standards between countries are crucial for the global adoption and commercial development of PSC technology, which can only be achieved through international cooperation. Furthermore, while balancing the need for the sharing of technology, transparent policies regarding intellectual property rights promote investment in research and development. There must be a regulatory framework in place to address possible concerns while also encouraging innovation in PSCs as they move toward commercialization. To foster an environment that encourages the sustainable expansion of PSC technology within a larger framework of clean and renewable energy adoption, it is essential that industry players, lawmakers, and regulatory agencies maintain continuous communication.

## 10. Conclusions

This exploration of PSCs has presented a collection of state-of-the-art advancements that have the potential to transform the field of photovoltaics. This review summarized the individual components of PSCs, offering a detailed understanding of their importance and potential and describing the efforts employed to overcome their limitations. Fueled by ongoing research and innovation, PSCs have become an important part of the renewable energy industry, demonstrating a rapid transformation from being a subject of scientific interest in laboratories to becoming a technology that is on the verge of being commercially viable. Their increasing significance is due to their exceptional characteristics, such as a high *PCE*, high adaptability, and the capacity to use a wide range of light wavelengths. 

This review summarized the present understanding of the layers that are essential to PSCs, including the absorbing layer, HTL, ETL, and different electrode types, highlighting the complex interactions between the various materials and their functions within different device designs. The absorbing layer, which is often made from OIHPs, is responsible for light absorption and charge creation, while the HTL and ETL assist in the movement of holes and electrons, respectively. The TCE and counter electrode are responsible for collecting charge carriers and completing the electrical circuit. Many approaches have been employed to enhance the performance of PSCs by focusing on optimizing the materials in these layers. In addition, the selection of materials has a substantial impact on the stability, efficiency, and environmental consequences of PSCs. The advantages of organic materials, such as their flexibility and ease of production, and the stability benefits of inorganic materials highlight the complexity inherent in PSC design and the importance of tuning the characteristics of active materials to obtain the optimal device performance. Design considerations also focus on underlying response processes, emphasizing the need for a comprehensive approach to material selection and device architecture.

Perovskite photovoltaics offer distinct characteristics that distinguish them from conventional solar systems. Their versatility enables seamless incorporation into a wide range of applications, from traditional solar panels to pliable and lightweight gadgets. The translucent nature and adaptability of PSCs allow them to be incorporated into architectural designs and visually appealing solar systems to be established. Their low weight makes them ideal for use in industries where weight is a crucial consideration, such as aircraft and portable electronics. Tandem perovskite–perovskite solar cells can also be used to overcome the efficiency constraints of single-junction cells by taking advantage of complementary bandgaps. Although there are difficulties in optimizing tandem structures, their ability to enhance the efficiency of PSCs highlights the versatility and adaptability of this technology. 

However, it is important to note that, although there have been notable advancements in improving the *PCE* and resolving stability issues, PSCs still face a number of limitations in relation to their commercialization, such as potential Pb toxicity. Thus, the search for alternative materials and processes continues in order to develop sustainable and eco-friendly energy solutions. Researchers and industry stakeholders are also involved in the development of manufacturing methods that can be easily scaled up, the establishment of testing standards, and ensuring compliance with regulatory frameworks. These efforts have the potential to produce PSCs that are an economically feasible and universally embraced energy alternative. 

PSCs can be considered an important intersection of materials science, engineering, and sustainable energy. The complex interactions between the components of a PSC highlight the need for joint research to attain optimal device performance and position PSCs as a fundamental component of a worldwide energy framework. PSCs have the potential to make clean energy more accessible due to their versatility, high efficiency, and attractive appearance. In this sense, PSCs represent not only a remarkable scientific advancement but also a promising symbol of progress toward a more sustainable and energy-efficient future.

## Figures and Tables

**Table 1:** Tolerance and octahedral factors hybrid perovskite structures. Reprinted from [34], Copyright (2016) by Royal Society of Chemistry.

Compounds	Tolerance Factor (*t*)	Octahedral Factor (m)
MADyI_3_	0.97	0.44
FADyI_3_	1.06	0.44
MASmI_3_	0.93	0.50
FASmI_3_	1.01	0.50
EASmI_3_	1.05	0.50
ACSmI_3_	1.06	0.50
MATmI_3_	0.98	0.43
FATmI_3_	1.06	0.43
MAYbI_3_	0.98	0.43
MACaI_3_	0.99	0.42
MASrI_3_	0.92	0.53
FASrI_3_	1.00	0.53
EASrI_3_	1.04	0.53
ACSrI_3_	1.05	0.53

**Table 2:** Examples of light harvesters for PSCs.

Active Layer Type			
Mixed Perovskite Structure		Double Perovskite Structure	
(PEA)_2_(MA)_2_[Pb_3_I_10_] (FA)_x_(MA)_1-x_PbI_3_ FA_x_Cs_1-x_PbI_3_ (FA)_x_(MA)_1-x_SnI_3_ FA_1-x_Cs_x_PbBr_3_ FA_x_PEA_1-x_PbI_3_ MA_1-x_DMA_x_PbI_3_ Cs_x_MA_y_DMA_z_PbI_3_ Cs_x_MA_1-x_PbBr_3_ MAPb(I_1-x_Br_x_)_3_ FAPb(I_1-x_Br_x_)_3_ CsPb(I_1-x_Br_x_)_3_ MAPbI_3-x_(BF_4_)_x_ FAPb_1-__x_Sn_x_I_3_ FA_1-x_MA_x_Pb(I_1-y_Br_y_)_3_ (FAPbI_3_)_1-x_(MAPbBr_3_)_x_ Pb-Halide	[58] [59] [60] [61] [62] [63] [62] [64] [65] [66] [67] [68] [69] [70] [71] [72] [73]	(MA)_2_TlBiBr_6_ MA_2_AgBiI_6_ (MA)_2_KGdCl_6_ MA_2_AgBiBr_6_ Cs_2_AgSbBr_6_ Cs_2_AgBiBr_6_ Cs_2_BiAgCl_6_ Cs_2_InAgCl_6_ Cs_2_InAgBr_6_	[69] [74] [75] [76] [77] [78] [79] [80] [81]

**Table 3:** Comparison of various compounds used in different layers of perovskite solar cells and their efficiencies.

Perovskite Layers	Compounds	Materials	Effieiciency	Reference
Perovskite Absorbing Layers	Halide Compounds	CH_3_NH_3_PbI	3.13%	[35]
	Halide Compounds	CH_3_NH_3_PbBr_3_	3.81%	[36]
	Organo-Lead Halide	Spiro-OMeTAD	10.00%	[38]
Hybrid Perovskites Absorbing Layers	Cesium Lead Halide	CsPbX_3_, X = I, Br, Cl	7.81%	
	Alternative Photosensitive Material	Guanidinium	19.64%	[54]
	Kesterite Family Materials	CZTS/CZTSe	10.19%	[111]
Hole Transport Layer	Organic HTL Organic HTL	Spiro-OMeTAD	25.21%	[173]
		PTAA	20.45%	[97]
		PEDOT:PSS	Low *V_oc_*	[101]
		PEDOT:PSS/V_2_O_5_	4.40%	[96]
		P3HT	Low efficiency	[174]
		P3HT/graphene	13.82%	
		P3HT/Cs_3_Sb_2_I_9_	2.5%	
		P3HT/Triphenylamine group	22.87%	[109]
	In Organic HTL	NiOx HTL	15.73%	[112]
		Cu-doped NiO_x_	17.81%	[116]
		Phthalocyanine (Pc)-doped NiO_x_	21.68%	[117]
		NiCo_2_O_4_	18.23%	[119]
		Mg doped NiCo_2_O_4_	16.71%	[128]
		CuO_x_ HTL	17.1%	
		Cr_2_O_3_@GP	18.5%	[144]
		Cr_2_O_3_@CNT	26.8%,	
		MoO_3_	19%	
ETL	Organic ETL	PCBM	18.3%	[147]
		PCBM/C60	19.84%	[148]
	Inorganic ETL	TiO_2_/Acetonitrile	10.2%	[149]
		TiO_2_/Ionic Liquid	7.3%	
		SnO_2_	17%	[152]
		ZnO/MAPbI_3_	18.01%	[157]
		ZnO/Nb_2_O_5_	16.86%	[158]
		ZnO/SnSe	27%	[159]

**Table 4:** Comparison of various additive components, passivation materials, transmission materials, and modified interface structures employed in perovskite solar cells [175,176].

Perovskite Components	Name of the Components	V_oc_ (V)	J_sc_ (mA/cm^2^)	FF (%)
Additive Components	MAPbI_3_/Pyrrole	1.142	22.38	75.20
	CsPbI_3_/Ethanol-MACL	0.928	20.75	68.43
	CH_3_NH_3_PbI_3_/Carbon Quantum Dots	0.94	22.35	51.27
	CsPbI_3_/[Zn(C_6_F_5_)_2_]	1.12	20.67	81.98
	CsPbI_2_Br/HEMA	1.23	15.81	82.98
Passivation Materials	(FAPbI_3_)_1-x_(MAPbBr_3_)_x_	1.18	25.20	78.40
	CH3NH_3_PbI_3_	1.15	22.51	77.33
	FASnI_3_	0.73	22.82	72.00
	MA_0.85_FA_0.15_PbI_3_	1.12	24.47	76.87
	FAPbI_3_	1.16	23.95	83.70
Transmission Material	Nb_2_O_5_	1.454	5.64	70.00
	In_2_S_3_	1.090	7.76	65.94
	MPA-BTTI	1.120	23.23	81.40
	BaTiO_3_/TiO_2_	0.970	20.50	65.00
	NH_2_-ZnO@SnO_2_	1.139	25.22	78.68
	ZTO-ZnS	1.150	23.80	77.70
Modified Interface structure	FTO/bl-RiO_2_/MAPbI_3_/PbS/Spiro-OMETAD/Au	1.140	23.17	72.83
	FTO/PCBM/perovskite/Spiro-OMETAD/Au	1.124	22.77	79.00
	ITO/SnO_2_/perovskite/PTAA/Metal	1.110	24.06	75.81
	FTO/c-TiO_2_/perovskite/PTABr/PTAA:LAD/Au	1.093	23.28	79.13
	ITO/NiO_x_/PTAA/(MAPbI_3_)_0.95_(MAPbBr_2_Cl)_0.05_/PCBM/BCP/Ag	1.190	22.23	81.71
	ITO/SnO_2_/MAPbI_3_/_4_-CIBA Spiro-OMETAD/Ag	1.160	22.76	74.00
	FTO/TiO_2_/perovskite/(Me-PDA)Pb_2_I_6_/Spiro-OMETAD/Au	1.130	24.61	79.00
	FTO/SnO_2_/BCP/Spiro-OMETAD/Ag	1.140	23.50	77.10
	mp-TiO_2_/MASnI_3_/spiro-MeOTAD	0.88	16.8	42.89
	mp-TiO_2_/MASn_x_Pb(1-x)I_3_/P_3_HT	0.42	20.04	50.01
	FAPbI_3_/spiro-MeOTAD	0.94	23.3	65.01
	MAPbI_3-x_Cl_x_/P_3_H	0.921	20.8	54.67
	ZnO nanorod/MAPbI_3_/spiro-MeOTAD	1.02	16.98	51.01
	mp-TiO_2_/MAPbI_3_/CuSCN	1.06	19.7	62.08

## Data Availability

Data is contained within the article.
